# A sensor array for the discrimination of polycyclic aromatic hydrocarbons using conjugated polymers and the inner filter effect†
†Electronic supplementary information (ESI) available. See DOI: 10.1039/c9sc03405f


**DOI:** 10.1039/c9sc03405f

**Published:** 2019-10-07

**Authors:** Joshua Tropp, Michael H. Ihde, Abagail K. Williams, Nicholas J. White, Naresh Eedugurala, Noel C. Bell, Jason D. Azoulay, Marco Bonizzoni

**Affiliations:** a Center for Optoelectronic Materials and Devices , School of Polymer Science and Engineering , The University of Southern Mississippi , 118 College Drive #5050 , Hattiesburg , MS 39406 , USA . Email: jason.azoulay@usm.edu; b Department of Chemistry and Biochemistry , The University of Alabama , P.O. Box 870336 , Tuscaloosa , AL 35487 , USA . Email: marco.bonizzoni@ua.edu

## Abstract

Natural and anthropogenic activities result in the production of polycyclic aromatic hydrocarbons (PAHs), persistent pollutants that negatively impact the environment and human health. Rapid and reliable methods for the detection and discrimination of these compounds remains a technological challenge owing to their relatively featureless properties, structural similarities, and existence as complex mixtures. Here, we demonstrate that the inner filter effect (IFE), in combination with conjugated polymer (CP) array-based sensing, offers a straightforward approach for the quantitative and qualitative profiling of PAHs. The sensor array was constructed from six fluorescent fluorene-based copolymers, which incorporate side chains with peripheral 2-phenylbenzimidazole substituents that provide spectral overlap with PAHs and give rise to a pronounced IFE. Subtle structural differences in copolymer structure result in distinct spectral signatures, which provide a unique “chemical fingerprint” for each PAH. The discriminatory power of the array was evaluated using linear discriminant analysis (LDA) and principal component analysis (PCA) in order to discriminate between 16 PAH compounds identified as priority pollutants by the US Environmental Protection Agency (EPA). This array is the first multivariate system reliant on the modulation of the spectral signatures of CPs through the IFE for the detection and discrimination of closely related polynuclear aromatic species.

## Abstract 2

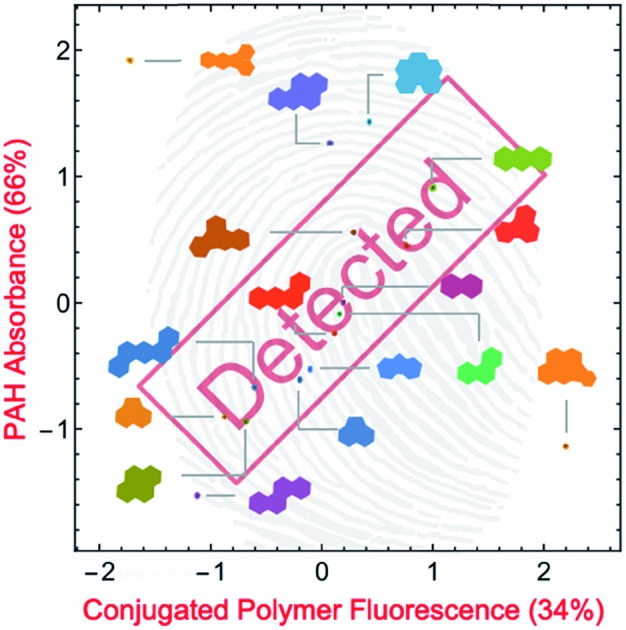
The inner filter effect and multivariate array sensing using conjugated polymers are combined for the detection and challenging discrimination of closely related polycyclic aromatic hydrocarbons.

## Introduction

Polycyclic aromatic hydrocarbons (PAHs) are a ubiquitous and prominent class of organic compounds comprised of fused aromatic rings containing only carbon and hydrogen. Well over 120 years of research has intricately connected these compounds with their natural and anthropogenic origins.^1^ While natural sources include those such as fossil fuels, open burning, and volcanic activity; pyrogenic and petrogenic sources such as the combustion of these fossil fuels, industrial manufacturing, and dispersed sources (*i.e.* automotive emission, residential heating, food preparation, *etc.*) predominate.^2^ These activities result in the production of PAHs that are pervasive environmental pollutants with toxic, mutagenic, and carcinogenic properties.^3^ For these reasons, research efforts remain unabated toward the detection and discrimination of these compounds; however, this continues to represent a major technological hurdle owing to their uncharged and nonpolar nature, similar and relatively featureless structures, lack of heteroatoms or substituents, and low active concentrations.^4^

Current methods for PAH detection necessitate sample collection, transport, protracted multi-stage extraction, preconcentration, and separation procedures. Subsequently, specialized analytical instrumentation sensitive to subtle chemical differences and employing multiple detectors is applied for analysis. Examples include high-performance liquid chromatography (HPLC) coupled to fluorescence or ultraviolet detection,^5^ gas chromatography coupled to mass spectrometry (GC-MS) or flame ionization detection (FID),^6^ and capillary electrophoresis coupled to fluorescence or ultraviolet detection.^7^ While highly sensitive, these methods require trained personnel, long analysis time, may lead to low analytical precision and analyte bias, are intended for a specific PAH type or property, and cannot discriminate between closely related compounds such as benzo[*a*]pyrene and dibenz[*a*,*h*]anthracene; isomers differing only in the connectivity of an aromatic ring and classified as carcinogens by numerous international agencies.^3^ Immunoassay-based approaches utilize antibody–antigen interactions, which demonstrate improved sensitivities in platforms that offer reduced costs, rapid detection, portability, and are amenable to high-throughput.^4^,^8^ Despite these advantages, similarities in the molecular and electronic structure of PAHs preclude the development of specific antibodies, leading to cross-reactivity and the inability to differentiate between closely related compounds.^9^ In a similar manner, various nanomaterial-based sensor platforms utilizing quantum dots, graphene, carbon nanotubes, and polymer composites have been advanced as alternative approaches for the detection of PAHs, but are fundamentally limited in their discriminatory power in multi-analyte detection.4c,^10^ As such, important questions regarding source attribution, transport and fate in the environment, bioaccumulation in the food chain, and the toxicology of these ubiquitous pollutants remain largely unresolved.^11^

Consequently, there remains an urgent need for the qualitative and quantitative assessment of complex mixtures of PAHs in a rapid, portable, high-throughput and cost-effective manner. Fluorescent chemosensory devices remain a leading signal transduction method owing to their high sensitivity, ease of operation, and broad applicability.^12^ When compared to small molecule fluorophores, significant gains in sensitivity are achieved using conjugated polymers (CPs) since their delocalized electronic structure leads to large extinction coefficients, strong fluorescence emission, efficient excited state (or exciton) migration, and collective properties that are sensitive to minor perturbations.^13^ In general, fluorescent sensors based on CPs operate through analyte-induced energy transfer, various aggregation phenomena, or conformational rearrangements that serve to manipulate mobile excitons and modulate the fluorescence in the form of spectral shifts, quenching, or unquenching of the emission.^12^,^14^ These mechanisms are distance-dependent and require strong CP-analyte interactions that are typically facilitated through the integration of molecular recognition elements (receptors) within or extended from the CP backbone.^15^ Nonspecific receptors for PAHs based on cation···π, relatively weak π···π and C–H···π interactions have been reported, but only show affinity toward particular PAHs.^16^ Supramolecular host–guest systems based on phenylene-bridged 4,4′-bipyridinium cations have demonstrated significant enhancements in binding affinities for PAHs, however, complex molecular topologies are required to form selective inclusion complexes and tailored chemistries for distinct PAHs are currently unavailable.^17^ Furthermore, the challenge of electronically coupling these analyte–receptor interactions into transducible optical responses further complicates the development of optical sensing platforms capable of profiling the distinct molecular signatures of diverse PAHs in complex mixtures.

More recently, array-based sensing has been used to profile combinations of structurally and chemically similar analytes through multivariate pattern recognition.^18^ Subtle structural differences between nonspecific CP-based sensing elements allow for differential interactions with analytes that establish unique and identifying optical response patterns.^19^ This creates a “chemical fingerprint” which can be used to discriminate similar compounds using linear discriminant analysis (LDA) and principal component analysis (PCA), pattern recognition algorithms which highlight and summarize distinguishing features in large data sets to provide information leading to chemical differentiation.^20^ Still, these methods require spatially distinct sensor units, each with its own recognition element, to build a diagnostic pattern that can be used to rapidly identify individual analytes. The inner filter effect (IFE) results from the absorption of light by a chromophore in solution, preventing photons from reaching a fluorophore, creating an observed decrease in fluorescence emission.^21^ Here, we demonstrate that the IFE in combination with CP-based array sensing offers a straightforward approach for the quantitative detection and qualitative discrimination of PAHs. While previous reports have demonstrated the utility of the IFE for the detection of picric acid and Sudan dyes using CPs,^22^ we report the use of differential quenching and pattern recognition to discriminate chemically and structurally similar PAHs, which could not be achieved using the IFE or CPs independently. To obtain the desired differential interactions required for an effective array sensor, we synthesized a series of similar but structurally distinct fluorescent CPs based on fluorene copolymer scaffolds with 2-phenylbenzimidazole optical modifiers. These CPs provide spectral overlap in regions of maximum absorption for many PAHs allowing for an IFE, with the PAH acting as optically dense absorbers. The reported system thus takes advantage of the intrinsic optical properties of individual PAHs, circumventing the need for tailored host–guest interactions. The unique response of each polymer allowed for the discrimination of 16 PAHs listed by the EPA as priority pollutants that are hazardous to human health.

## Results and discussion

### Polymer design, synthesis, and optical characterization

**Fig. 1:**
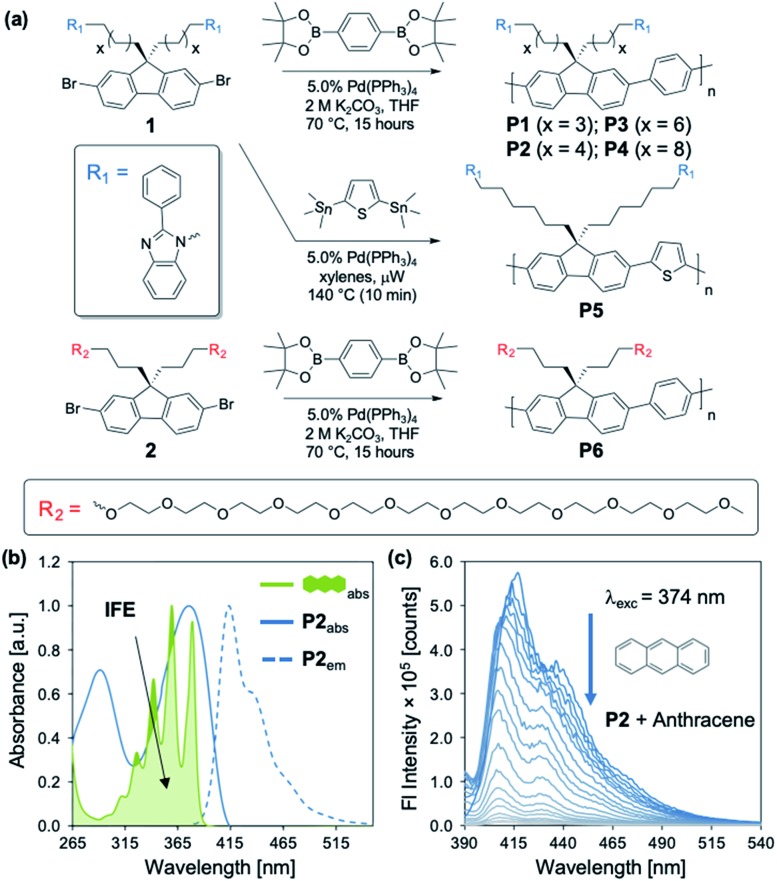
(a) Synthesis of **P1–P6**. (b) Normalized excitation and emission spectra of **P2** overlaid with normalized anthracene absorption ([anthracene] = 10 μM; [**P2**] = 15 mg L^–1^), providing the basis for an IFE. (c) Fluorescence spectra of **P2** (15 mg L^–1^) upon titration with anthracene (0–9.4 mM) in *N*,*N*-dimethylformamide (DMF) (*λ*_exc_ = 374 nm).

**Fig. 2:**
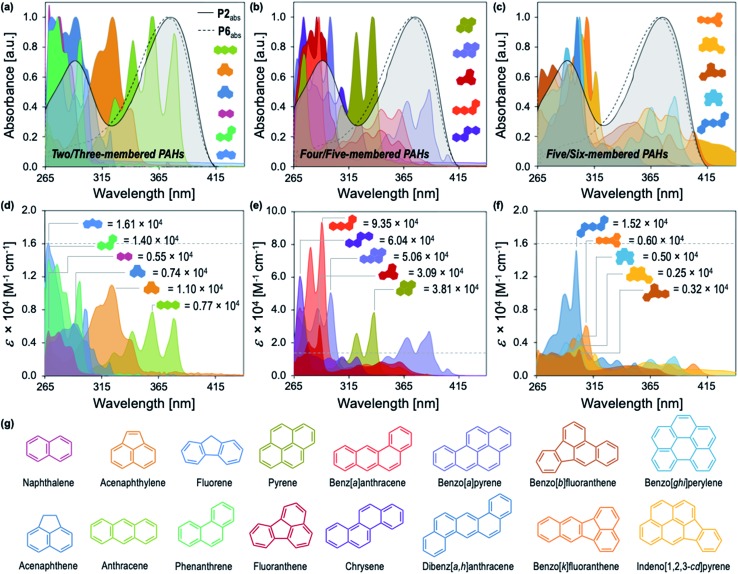
Normalized UV-vis absorption spectra of **P2** (solid line, [**P2**] = 15 mg L^–1^) compared to **P6** (dashed line, [**P6**] = 15 mg L^–1^) overlaid with the normalized absorption spectra of (a) two- and three-membered PAHs, (b) four- and five-membered PAHs, and (c) five- and six-membered PAHs in DMF ([PAHs] = 10 μM). Wavelength dependence of *ε* for (d) two- and three-membered PAHs, (e) four- and five-membered PAHs, and (f) five- and six-membered PAHs in DMF. Extinction coefficients at *λ*_max_ for each PAH are annotated. (g) The structures of all 16 PAHs identified by the EPA as priority pollutants.

Our studies began with the synthesis of poly[2,7-(9,9-di((6′-(2-phenyl-1*H*-benzo[*d*]imidazole))hexyl)-fluorene)-*alt*-1,4,-phenylene] (**P2**) (Fig. 1a) using a Suzuki cross-coupling polymerization.^23^ We utilized the same approach for the synthesis of **P1–P4** and **P6**, while **P5** was synthesized using a microwave mediated Stille cross-coupling polymerization (Fig. 1a). These approaches resulted in facile access to an array of structurally distinct polymers, that were targeted to maintain solubility, high fluorescence emission, and sizes beyond the exciton diffusion length.^24^ General protocols regarding monomer and polymer synthesis are included in the Experimental section with full details in the ESI.† **P2** exhibits an absorption maximum (*λ*_max_) centered at 374 nm, providing spectral overlap in regions of maximum absorption for many PAHs (Fig. 1 and 2). Each PAH displays a characteristic absorption profile in the 250–500 nm region (Fig. 2 and S2–S17†). The tunable nature of these CPs allows for the incorporation of optical modifiers which provide greater spectral overlap between the absorption of the polymer and each PAH, enabling efficient fluorescence quenching through the IFE. Peripheral 2-phenylbenzimidazole substituents in **P2** impart an extra absorption band with *λ*_max_ = 290 nm, which affords the greater spectral overlap required for the detection of PAHs through the IFE (Fig. S18†). Fig. 1b illustrates the significant spectral overlap between **P2** and anthracene allowing for an IFE, with the PAH acting as a “chemical filter.” Upon excitation at 374 nm, **P2** exhibits a strong emission between 400–500 nm, with maximum intensity at 415 nm. Addition of anthracene causes apparent quenching of the fluorescence through the IFE (Fig. 1c).

Fig. 2 illustrates the distinct optical profiles of the 16 PAH compounds identified as priority PAH pollutants by the US Environmental Protection Agency (EPA). Each PAH demonstrates varying spectral overlap with **P2** (Fig. 2a–c), and distinct wavelength dependencies of the molar absorptivity (*ε*), providing the basis for differential quenching through the IFE (Fig. 2d–f). To obtain the desired diversity of interactions required for an effective array sensor, a series of similar but structurally distinct CPs were synthesized, which demonstrate fluorescence quenching by PAHs through the IFE (Fig. 1a). In addition to **P2**, the polymer array included poly[2,7-(9,9-di((5′-(2-phenyl-1*H*-benzo[*d*]imidazole))pentyl)-fluorene)-*alt*-1,4,-phenylene] (**P1**), poly[2,7-(9,9-di((8′-(2-phenyl-1*H*-benzo[*d*]imidazole))octyl)-fluorene)-*alt*-1,4,-phenylene] (**P3**), poly[2,7-(9,9-di((10′-(2-phenyl-1*H*-benzo[*d*]imidazole))decyl)-fluorene)-*alt*-1,4,-phenylene] (**P4**), poly[2,7-(9,9-di((6′-(2-phenyl-1*H*-benzo[*d*]imidazole))hexyl)-fluorene)-*alt*-2,5,-thiophene] (**P5**), and poly[2,7-(9,9-di((undec(ethylene glycol)monomethyl ether))-fluorene)-*alt*-1,4,-phenylene] (**P6**).

**Fig. 3:**
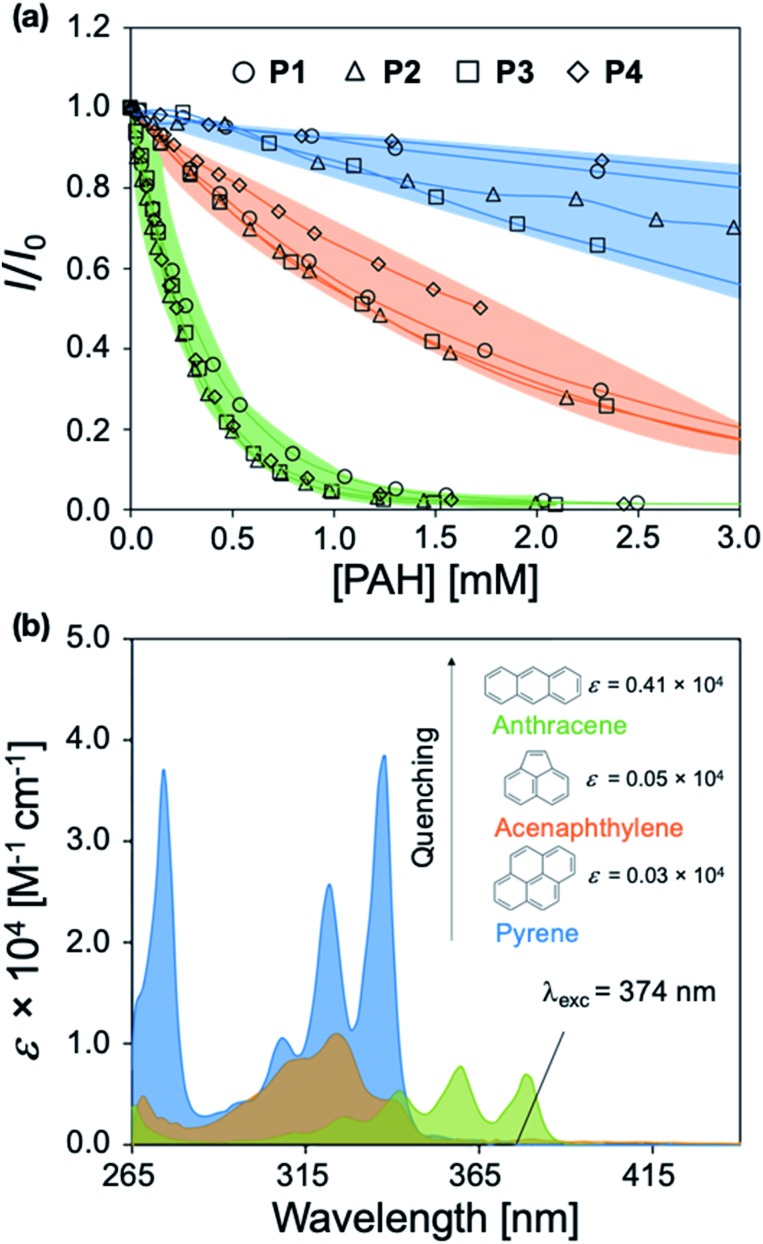
(a) Fluorescence titration profiles of **P1–P4** upon the addition of aliquots of anthracene, acenaphthylene, and pyrene in DMF (*λ*_exc_ = 374 nm/*λ*_em_ = 418 nm). (b) Wavelength dependence of the molar extinction coefficient for anthracene, acenaphthylene, and pyrene. The colors indicate the PAH involved.

**P1–P4** demonstrate a systematic lengthening of the methylene bridge (–CH_2_–)_*x*_ between the 2-phenylbenzimidazole optical modifier and the CP backbone, with *x* = 3, 4, 6, and 8 units, respectively (Fig. 1a). The structural changes in the polymer series cause subtle but distinct differences in their optical spectra and molar absorptivity (Fig. S1†). To establish that these slight modifications could manifest into discernible responses, several PAHs were titrated into solutions of **P1–P4** to study their effect on the fluorescence emission of each polymer. Three PAHs, anthracene, acenaphthylene, and pyrene were chosen to represent a range of ring fusion and optical properties (*e.g.* intrinsic absorption) in the PAH family. The resulting titration profiles for **P1–P4** are summarized in Fig. 3a, in which quenching of polymer emission is caused by the selected PAHs through the IFE. Small but noticeable differences in quenching were observed between **P1–P4** and each PAH, demonstrating that even subtle structural modifications affected the spectral response. More dramatic differential responses were shown between the PAHs, which can be explained by the distinct dependence of molar absorptivity for each PAH at a given wavelength (Fig. 3b). At an excitation wavelength (*λ*_exc_) of 374 nm, anthracene has the greatest molar extinction coefficient (*ε* = 0.41 × 10^4^ M^–1^ cm^–1^) and was the most efficient fluorescence quencher of each polymer through the IFE. As a representative example, the detection limit of anthracene using **P2** was calculated to be 2.4 μM, demonstrating the low limit of detection (LOD) for the array (Fig. S19†). A fluorene copolymer with a thiophene structural unit in the backbone (**P5**) was incorporated into the array to provide distinctive quenching behavior from the other copolymers. **P5** shows a red-shifted absorption (*λ*_max_ = 420 nm) and allows for differential quenching through the IFE when compared to the other copolymers of the array. A fluorene-*co*-phenylene copolymer without 2-phenylbenzimidazole optical modifiers (**P6**) and incorporated oligo(ethylene glycol) side chains was synthesized. **P6** lacks the extra band in the absorption spectrum (*λ*_max_ = 290 nm) seen in **P1–P5**, providing another source of differential interaction through the IFE. Minor structural variations between each polymer, in combination with the unique relationship of molar absorptivity and wavelength for each PAH, provide a library of differential responses which can be used for discrimination.

### Conjugated polymer-based sensor array for polycyclic aromatic hydrocarbons *via* pattern recognition

Solutions of **P1–P6** in DMF (15 mg L^–1^) were arranged on a 384-well plate and exposed to 500 μM solutions of each PAH (Fig. 2g).^25^ Each PAH-polymer combination was prepared in 12 replicates and multiple spectroscopic measurements were collected on a microwell plate reader, corresponding to the regions of spectral overlap between the polymers and each PAH, including 21 absorbance measurements from 280–700 nm, and fluorescence measurements using the following filter combinations (*λ*_exc_/*λ*_em_): 330/460 nm, 330/485 nm, 330/516 nm, 380/460 nm, 380/485 nm, and 380/516 nm. The raw instrumental response pattern for each measurement is tabulated and visually summarized as a heat map in the ESI (Fig. S20–S40†).

**Fig. 4:**
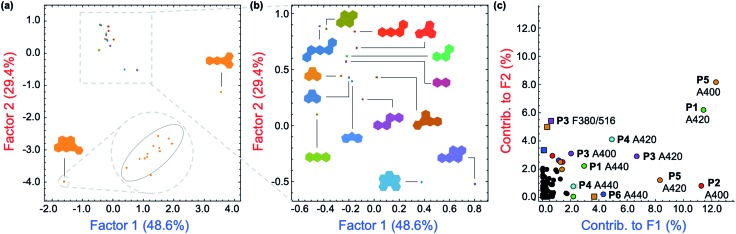
(a) Two-dimensional plot of the LDA scores for the attempted differentiation of 16 PAHs with **P1–P6**. The plot was generated using 114 instrumental variables and captures 78.0% of the total information in the raw dataset. The inset shows a representative tight intra-cluster spacing of 12 replicate samples of indeno[1,2,3-*cd*]pyrene ([PAH] = 500 μM). (b) Zoomed-in LDA scores plot from (a) for 14 PAHs, displaying low inter-cluster spacing between those PAHs. (c) LDA loadings plot for the differentiation of 16 PAHs shown in (a), indicating the relative contributions of each instrumental variable to the first two LDA factors. Colored squares: contributions from fluorescence measurements. Colored circles: contributions from absorbance measurements.

Superfluous instrumental variables introducing experimental noise were removed from the original data set whose contribution to PAH discrimination was negligible; these included absorbance measurements above 500 nm as PAHs lack absorption in this region. After removal of these absorbance measurements, the optical responses of **P1–P6** to each PAH were analyzed through linear discriminant analysis (LDA), a well-established multivariate patterning algorithm.^26^ Complete differentiation of 16 PAHs was observed using the first two factors obtained from LDA analysis, while retaining 78.0% of the total information content that was present in the raw dataset. Fig. 4a displays the corresponding two-dimensional LDA scores plot. Replicates of the same PAH sample were found to cluster tightly, whereas clusters of replicates from different samples were well-separated. Tight intra-cluster spacing indicates excellent reproducibility, while large inter-cluster spacing indicates strong discriminatory power of the polymer-based array.

The most important contributors to the differentiation were absorption signals in the range of 420–480 nm, as shown in the corresponding LDA loadings plot (Fig. 4c), which arise from absorption bands displayed only by benzo[*k*]fluoranthene and indeno[1,2,3-*cd*]pyrene. The unique features of these two PAHs between 420–480 nm are overrepresented in the discrimination displayed in Fig. 4a and are therefore assigned a disproportionally high weighting in the LDA analysis, thus differentiating these two very well from the other 14 PAHs, but providing very little discriminatory power for the other 14, thus drastically reducing the array's effectiveness for analytical applications. An overview of the quality of the information conveyed by each instrumental measurement is presented visually in the ESI (Fig. S42†). The absorbance measurements for each polymer between 420–480 nm contain little coherent signal and are dominated by noise, relative to the absorption at lower wavelengths (<420 nm) and were therefore removed from the dataset. The LDA analysis was then repeated on this reduced dataset.

**Fig. 5:**
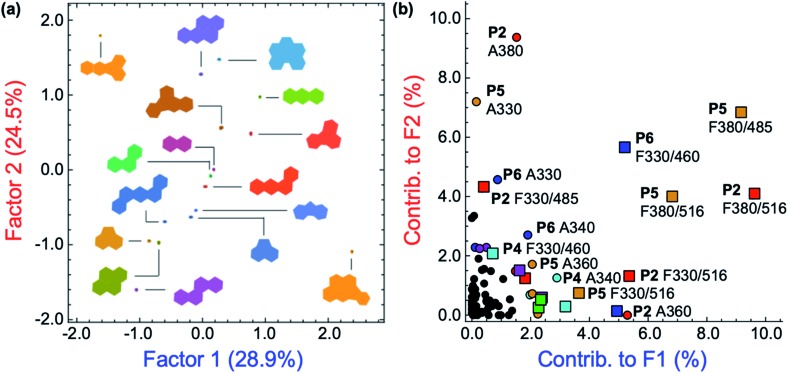
(a) The two-dimensional plot of the LDA scores for the differentiation of 16 PAHs with polymers **P1–P6** ([PAH] = 500 μM; [**P1–P6**] = 15 mg L^–1^). This plot was generated using the most important instrumental variables (79 in total) and captures 53.4% of the total information contained in the raw dataset. (b) LDA loadings plot for the differentiation of 16 PAHs shown in (a), indicating the relative contributions of each instrumental variable to the first two LDA factors. Colored squares: contributions from fluorescence measurements. Colored circles: contributions from absorbance measurements.

After removal of the information-poor absorbance measurements between 420–480 nm, subsequent LDA analysis and data reduction provided a better performing differentiation, representing a significant improvement in the overall discriminatory power of the array (Fig. 5a). The corresponding LDA loadings plot reflects a reduced importance of absorption signals to the differentiation, with a more diverse group of variables such as the fluorescence measurements acting as the most important contributors to the discrimination (Fig. 5b). Increased reliance on fluorescence signals is desirable as the high photoluminescence quantum efficiencies of fluorene-based copolymers should offer considerably lower limits of differentiation for PAHs compared to an array relying primarily on the absorption properties of PAHs. Despite the improvement in separation of all 16 PAHs, only half of the total information was retained in the first two factors (53.4%). By including a third factor, the data could be displayed as a three-dimensional plot, while preserving a larger portion of the total information. As shown in the ESI (Fig. S43†), the three-dimensional scores plot now retains 74.7% of the total information contained in the original dataset.

The dataset was also analyzed by a similar multivariate technique, principal component analysis (PCA), as PCA provides an unsupervised representation of the variances in a given dataset. The two-dimensional PCA plot is presented in Fig. S44 in the ESI.† PCA analysis also gives complete separation of all 16 PAHs with very small intra-cluster distances, while retaining 70.8% of the total information from the original dataset within the first two components.

### Role of the inner filter effect in polycyclic aromatic hydrocarbon discrimination

**Fig. 6:**
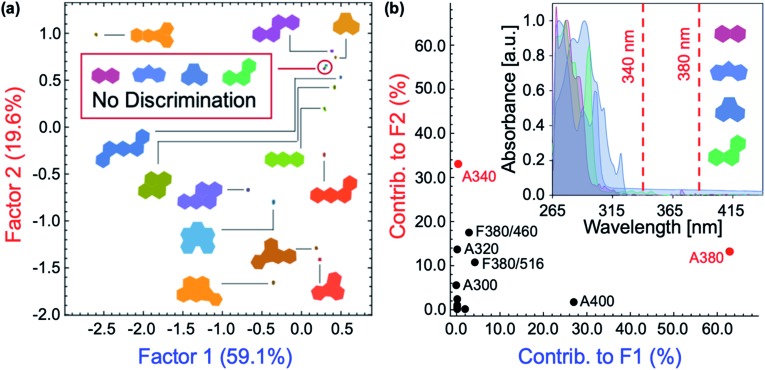
(a) LDA scores plot for the attempted differentiation of 16 PAHs in the absence of polymers at [analyte] = 500 μM. (b) Two-dimensional loadings plot for factors F1 and F2 in the linear discriminant analysis of 16 PAHs without polymers. Inset: UV-vis absorption spectra of naphthalene, fluorene, acenaphthene, and phenanthrene.

The role of **P1–P6** in the discrimination was investigated by analyzing a dataset containing optical measurements from only the PAHs, in the absence of polymers. LDA analysis of this dataset showed that complete separation of all 16 PAHs was not achieved (Fig. 6a). Moreover, the corresponding loadings for the first two factors (Fig. 6b) are, once again, mostly reliant upon the absorbance measurements of the PAHs: not only is this detrimental for the sensitivity of the assay, it also prevents PAHs with similar absorption features such as naphthalene, fluorene, acenaphthene, and phenanthrene from being differentiated (Fig. 6b, inset). The modulation of **P1–P6** fluorescence was therefore found to be critical in the discrimination of all 16 PAHs, as demonstrated by the large contribution of fluorescence measurements in the factor loadings (Fig. 5b).

**Fig. 7:**
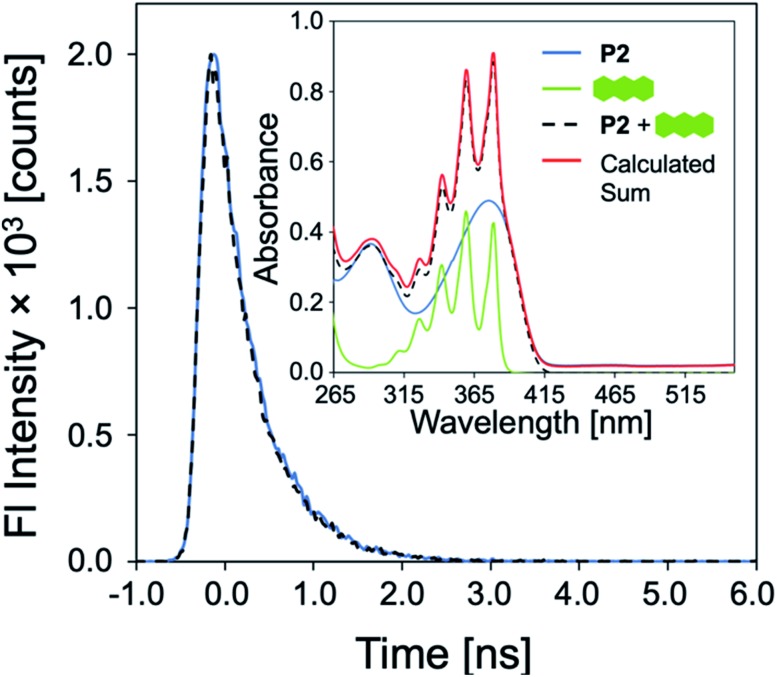
Emission decay of **P2** (15 mg L^–1^) before and after addition of anthracene (500 μM) in DMF. Inset: UV-vis absorption spectra of **P2**, anthracene, of a mixture of **P2** and anthracene, compared to a simulated spectrum of this mixture calculated from the sum of the individual experimental spectra.

To elucidate the mechanism of fluorescence quenching, steady-state absorption, fluorescence lifetime, and fluorescence anisotropy measurements were performed. As illustrated in Fig. 2, the PAH absorption spectra span 250–480 nm, overlapping with the absorption and emission spectra of **P1–P6**. The spectral overlap indicates the possibility of fluorescence resonance energy transfer (FRET),^27^ which may take place in the presence of overlapped emission spectrum of a fluorophore (CP) with the absorption spectrum of a quencher (PAH), or the IFE. The fluorescence lifetime was measured in the absence and presence of PAH, where **P2** and anthracene were chosen as a representative example. It is evident from Fig. 7 that the fluorescence lifetime of **P2** does not display any significant change upon the addition of anthracene, which rules out a dynamic quenching mechanism.^22^ Furthermore, the fact that the absorption spectrum of a mixture between polymer and PAH is completely additive rules out static quenching due to aggregation and formation of a ground–state complex between **P2** and anthracene (Fig. 7, inset). Fluorescence anisotropy experiments are commonly used to investigate molecular interactions between a small fluorophore and a macromolecule, where a strong increase in anisotropy indicates a slower rotational diffusion rate of the fluorophore due to binding.^27^,^28^ For these experiments, **P5** was titrated into a solution of anthracene ([anthracene] = 100 μM in DMF). Polymer **P5** was chosen because its emission spectrum is well-separated from that of anthracene, allowing the emission of anthracene and changes in its tumbling rate in solution to be directly observed. However, no increase in fluorescence anisotropy was observed (Fig. S45†), suggesting that no binding occurred between anthracene and **P5**. When all these results are considered, the IFE is the most probable mechanism responsible for quenching of the polymers.

## Conclusions

In summary, we have demonstrated the detection of 16 priority PAHs through a six-membered sensor array. A new set of fluorescent CPs was readily prepared, incorporating side chain optical modifiers, which were strongly and yet non-selectively affected by the presence of diverse PAHs through an IFE. Multivariate pattern recognition strategies were used to generate two-dimensional score plots, which can operate as effective calibration plots for the differentiation of unknown PAHs using simple, common, and cost-effective instrumentation (UV-vis absorption and fluorescence spectroscopies). The reported platform is highly tunable, owing to facile modification of the polymer structure and ease with which optical modifiers can be designed and integrated. The discrimination of similar analytes that lack well-defined recognition elements was achieved, widening the scope of the IFE in CP-based sensors. These results lay the groundwork for an extension into qualitative and quantitative detection and discrimination of molecular species with high optical densities that are otherwise inaccessible through energy transfer and aggregation-based mechanisms traditionally utilized in CP-based array sensors.

## Experimental section

### Materials

Reagents were purchased from Sigma-Aldrich and used without further purification, unless otherwise specified. Spectral grade *N*,*N*-dimethylformamide (DMF) was purchased from EMD Chemicals Inc. and used as received. Xylenes and tetrahydrofuran (THF) were degassed and dried over 4 Å molecular sieves. Chloroform-*d* (CDCl_3_) was purchased from Cambridge Isotope Labs and used as received. Tetrakis(triphenylphosphine)palladium(0) (Pd(PPh_3_)_4_) was purchased from Strem Chemicals and used without further purification. 2,7-Dibromo-9,9-bis(5-bromopentyl)-9*H*-fluorene,^29^ 2,7-dibromo-9,9-bis(8-bromooctyl)-9*H*-fluorene,^30^ and 1,1′-((2,7-dibromo-9*H*-fluorene-9,9-diyl)bis(hexane-6,1-diyl))bis(2-phenyl-1*H*-benzo[*d*]imidazole)^23^ were prepared according to literature procedures.

### General procedure for the synthesis of functionalized monomers

2,7-Dibromo-9,9-bis(6-bromoalkyl)-9*H*-fluorene (1.02 mmol, 1.00 equiv.), 2-phenylbenzimidazole (2.24 mmol, 2.20 equiv.) and NaH (4.08 mmol, 4.00 equiv.) were dissolved in DMF (10 mL) under nitrogen. The mixture was stirred at 70 °C for 12 h. The reaction mixture was then cooled to room temperature, quenched with deionized water, and extracted with ethyl acetate (3 × 50 mL). The organic layer was then washed with water (3 × 50 mL) and dried over anhydrous MgSO_4_. Volatiles were removed *in vacuo*, and purification was accomplished by flash chromatography using a hexanes : ethyl acetate gradient.

### General procedure for the synthesis of functionalized conjugated polymers

A microwave tube was loaded with bifunctional aromatic bromide (**SI-1–SI-3**) (0.100 mmol, 1.00 equiv.), 1,4-bis(4,4,5,5-tetramethyl-1,3,2-dioxaborolan-2-yl)benzene (0.105 mmol, 1.05 equiv.), 2 M K_2_CO_3_ (1.0 mL, 20 equiv.) in water, and THF (1.0 mL). The mixture was sparged with nitrogen, and 0.70 mL of a Pd(PPh_3_)_4_/THF stock solution (5 mol%) was added *via* syringe. The mixture was stirred vigorously and heated at 70 °C for 15 h. After this time, the reaction was allowed to cool leaving a solid gelled material. The mixture was precipitated into methanol and collected *via* filtration. The residual solid was loaded into an extraction thimble and washed with methanol (8 h), acetone (4 h), and hexanes (4 h). The polymer was dried *in vacuo*.

### Spectroscopic methods

All spectra were recorded at ambient temperature, unless otherwise stated. UV-vis absorbance measurements were performed on a Hewlett-Packard 8452a diode array UV-vis spectrophotometer. Benchtop steady-state fluorescence measurements were carried out with an ISS PC1 spectrofluorimeter. Excitation was carried out using a broad-spectrum high-pressure xenon lamp (CERMAX, 300W). Excitation correction was performed through a rhodamine B quantum counter with a dedicated detector. Detection was through a Hamamatsu red-sensitive PMT. High-aperture Glan-Thompson calcite polarizers were used in the excitation and emission channels to measure steady-state fluorescence anisotropy. Experimental temperature (25 °C) was controlled by an external circulating water bath.

All optical spectroscopy and binding experiments were performed in DMF. Polymer and PAH stock solutions were prepared separately, then (2 mL) of the polymer solutions were inserted into a quartz cuvette, and absorption and fluorescence spectra were collected for **P1–P6**. Fluorescence emission spectra were collected by exciting the polymer at their most red-shifted spectral maximum. Binding titrations were performed by adding aliquots (1–100 μL per addition) of PAH solution to the polymers. Fluorescence decay profiles were recorded using a Horiba PPD850 time-correlated single-photon counting (TCSPC) detector with a Fianium WhiteLase SC-400 laser excitation source at a repetition rate of 2 MHz.

Multivariate data was acquired on a BioTek *Synergy II* multimode microwell plate reader, capable of measuring absorption spectra through a monochromator and steady-state fluorescence intensity measurements through a set of bandpass filters. The sample compartment in this instrument was electrically thermostatted to 25 °C. Experiments were laid out by hand using Eppendorf Research multichannel pipettors and disposable plastic tips into Aurora microwell plates with clear bottoms for UV absorption and fluorescence spectroscopy in a 384-well configuration. The plates were made of non-treated cyclo-olefin polymer (COP) with clear flat bottoms. Each well was filled with (100 μL) of the sample solution. Plates were read on a multimode plate reader immediately after preparation. Further details regarding multivariate data analysis are available in the ESI.†

## Conflicts of interest

There are no conflicts to declare.

## Supplementary Material

Supplementary information
