# Supplementary material for: A sensor array for the discrimination of polycyclic aromatic hydrocarbons using conjugated polymers and the inner filter effect
Source: Chem Sci. 2019 Oct 7;10(44):10247–55. doi: 10.1039/c9sc03405f (PMC7020785; doi:10.1039/c9sc03405f)
Supplement: Supplementary file 1 [file SC-010-C9SC03405F-s001.pdf]

Electronic Supplementary Material (ESI) for Chemical Science.
This journal is © The Royal Society of Chemistry 2019

Supplementary Information

A Sensor Array for the Discrimination of Polycyclic Aromatic Hydrocarbons Using Conjugated Polymers and the Inner Filter Effect

Joshua Tropp,[†] Michael H. Ihde,[‡] Abagail K. Williams,[†] Nicholas J. White,[‡] Naresh Eedugurala,[†] Noel C. Bell,[†] Jason D. Azoulay,[*,†] and Marco Bonizzoni [*,‡]

[†] Center for Optoelectronic Materials and Devices, School of Polymer Science and Engineering, The University of Southern Mississippi, 118 College Drive #5050, Hattiesburg, MS 39406, United States

[‡] Department of Chemistry and Biochemistry, The University of Alabama, P.O. Box 870336, Tuscaloosa, AL 35487, United States

Corresponding authors

***, ‡ marco.bonizzoni@ua.edu**

***, † jason.azoulay@usm.edu**

Table of contents:

1. General considerations...S-2
2. UV-Vis and fluorescence spectra for PAHs and **P1-P6** S-3
3. Multivariate data analysis S-23
4. Synthesis of functionalized monomers S-30
5. Synthesis of functionalized conjugated polymers.................................S-32
6. NMR Spectra S-36
7. References...S-42

1. General Considerations

All manipulations of air and/or moisture sensitive compounds were performed under an inert atmosphere using standard glove box and Schlenk techniques. ^{1}H and ^{13}C NMR spectra were collected on a Bruker Avance III 600 MHz spectrometer and chemical shifts, δ (ppm) were referenced to the residual solvent impurity peak of the given solvent. Data reported as: s = singlet, d = doublet, t = triplet, m = multiplet, br = broad; coupling constant(s), *J* are given in Hz. Flash chromatography was performed on a Teledyne Isco Combi*Flash* purification system using Redi*Sep* Rf prepacked columns. Microwave assisted reactions were performed in a CEM Discover microwave reactor. The number average molecular weight (M_n) and dispersity (*Đ*) were determined by gel permeation chromatography (GPC) relative to polystyrene standards at 160 °C in 1,2,4-trichlorobenzene (stabilized with 125 ppm of BHT) in an Agilent PL-GPC 220 High Temperature GPC/SEC system using a set of four PLgel 10 μm MIXED-B columns. Polymer samples were pre-dissolved at a concentration of 1.00 – 2.00 mg mL^{-1} in 1,2,4-trichlorobenzene with shaking for 2 h at 160 °C. High resolution mass spectra (HRMS) were recorded on a Bruker 12 Tesla APEX-Qe FTICR-MS with an Apollo ion source using positive-ion mode electrospray ionization.

2. UV-Vis and fluorescence data for PAHs and P1-P6

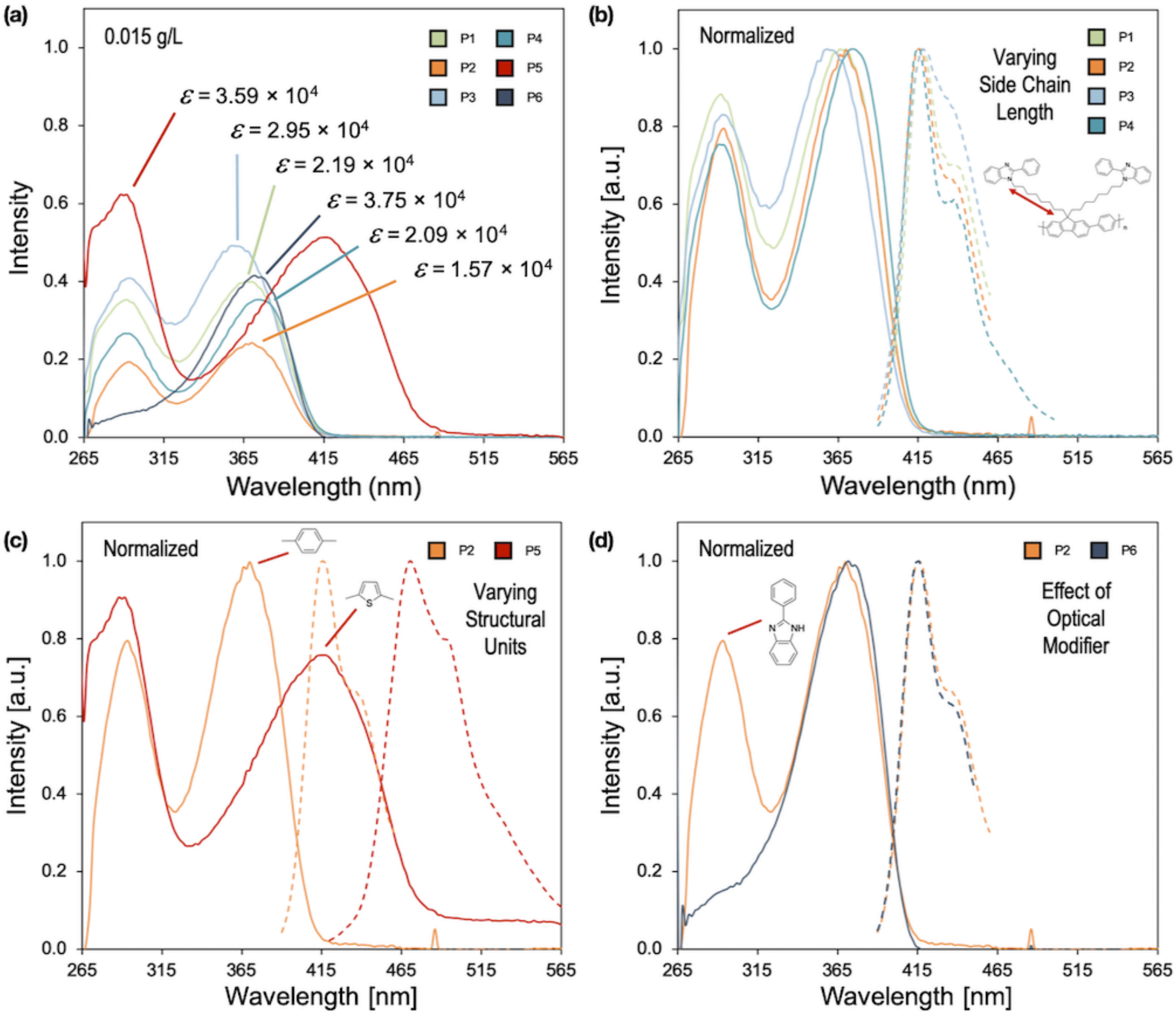

Fig. S1 (a) Absorption spectra of **P1-P6** at 15 mg L^{-1} in DMF. Extinction coefficients at λ_{max} for each polymer are annotated. (b) Normalized absorption and fluorescence spectra of **P1-P4** in DMF. (c) Normalized absorption and fluorescence spectra of **P2** and **P5** in DMF. (d) Normalized absorption and fluorescence spectra of **P2** and **P6** in DMF.

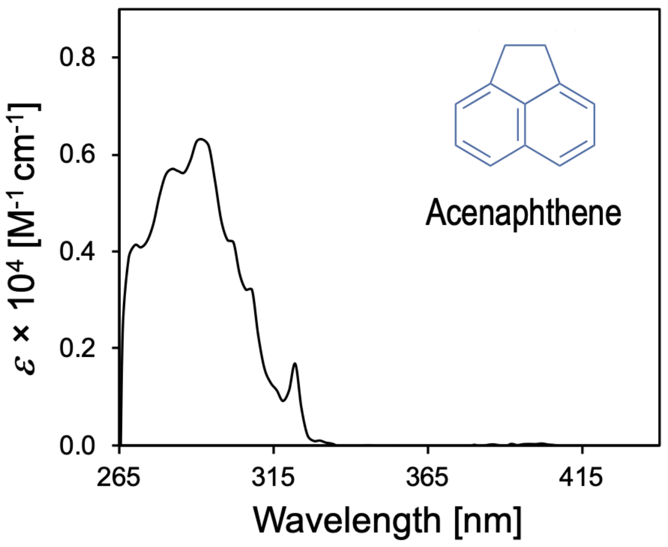

Fig. S2 Molar extinction spectra for acenaphthene (10 μM) in DMF. The fluorescence spectrum is not plotted as acenaphthene does not significantly absorb at λ_{exc} = 330 nm.

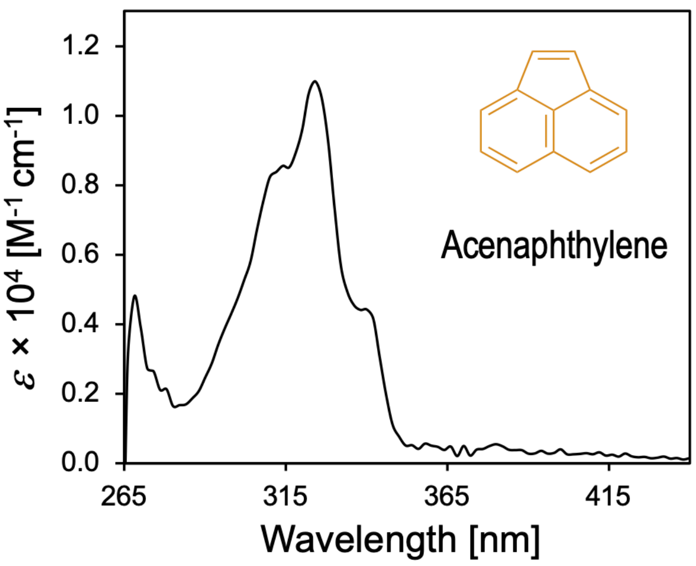

Fig. S3 Molar extinction spectra for acenaphthylene (10 μM) in DMF. The fluorescence spectrum is not plotted as acenaphthylene does not significantly absorb at λ_{exc} = 330 nm.

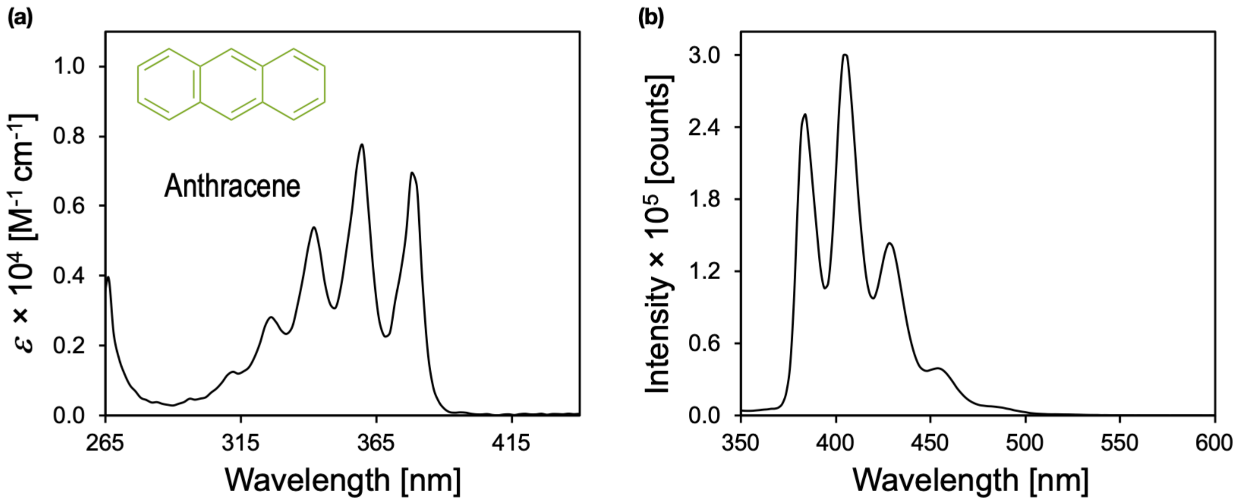

Fig. S4 (a) Molar extinction and (b) fluorescence (λ_{exc} = 330 nm) spectra for anthracene (10 μM) in DMF.

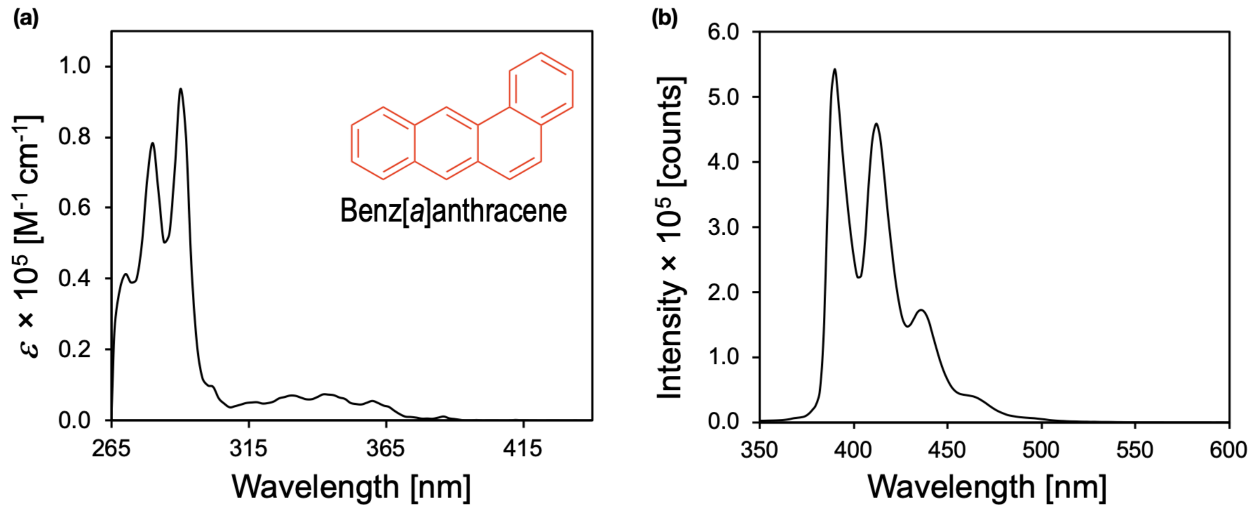

Fig. S5 (a) Molar extinction and (b) fluorescence (λ_{exc} = 330 nm) spectra for benz[*a*]anthracene (10 μM) in DMF.

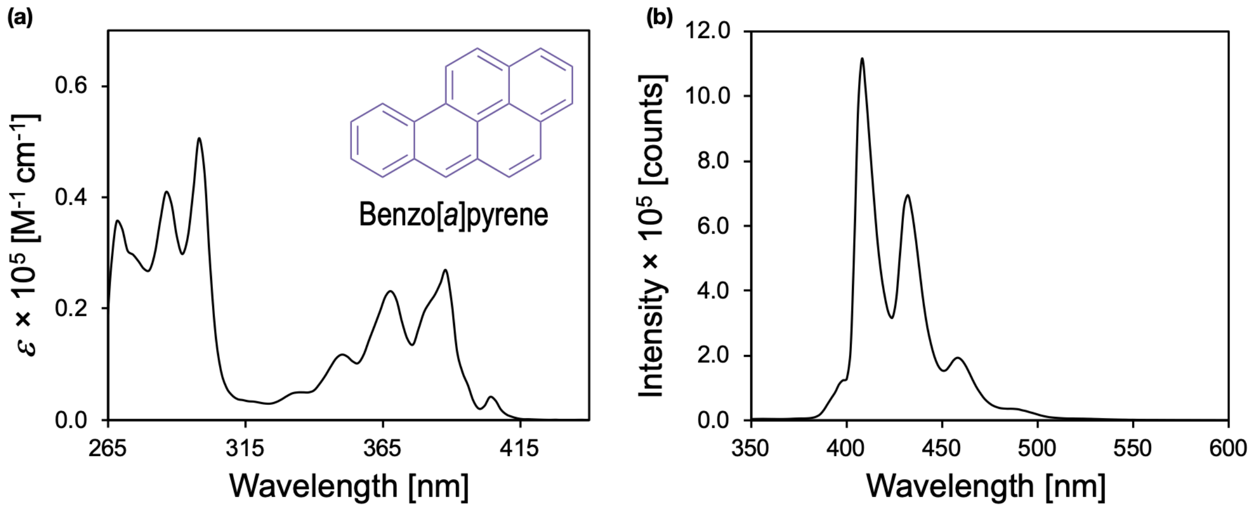

Fig. S6 (a) Molar extinction and (b) fluorescence (λ_{exc} = 330 nm) spectra for benzo[*a*]pyrene (10 μM) in DMF.

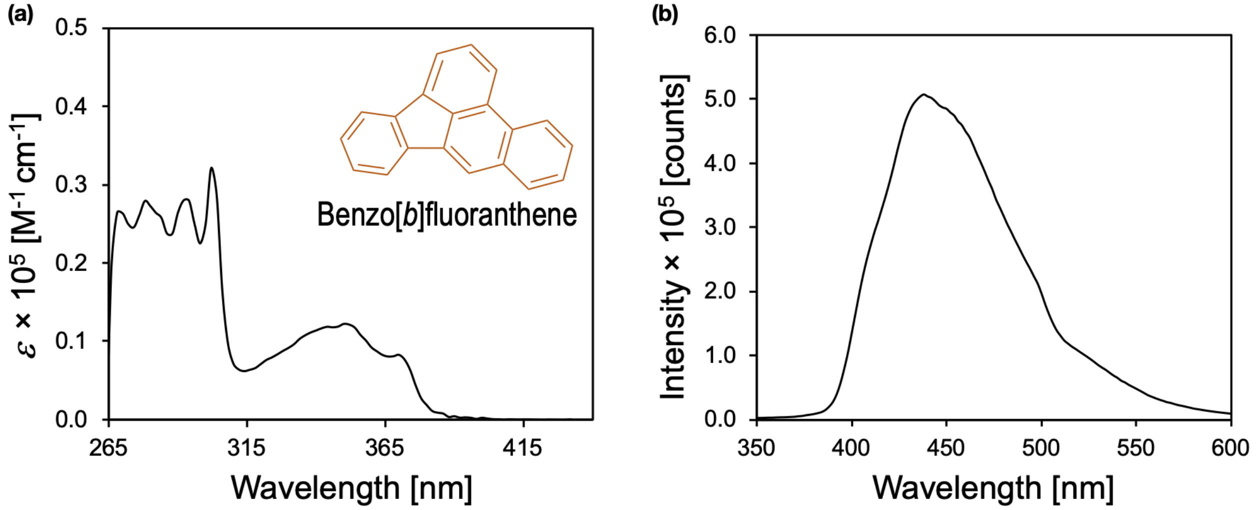

Fig. S7 (a) Molar extinction and (b) fluorescence (λ_{exc} = 330 nm) spectra for benzo[*b*]fluoranthene (10 μM) in DMF.

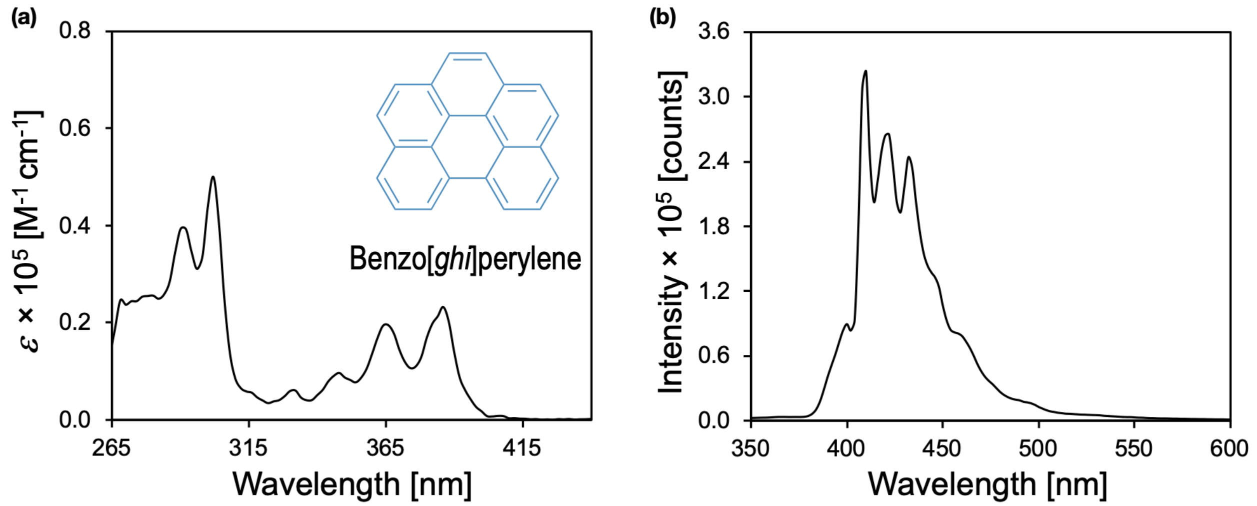

Fig. S8 (a) Molar extinction and (b) fluorescence (λ_{exc} = 330 nm) spectra for benzo[*ghi*]perylene (10 μM) in DMF.

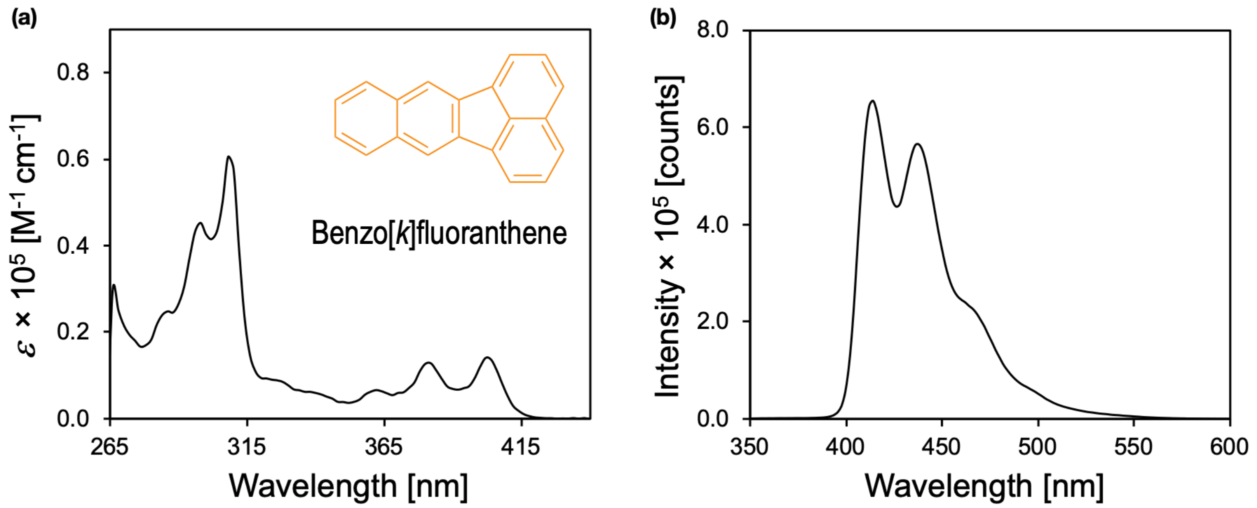

Fig. S9 (a) Molar extinction and (b) fluorescence (λ_{exc} = 330 nm) spectra for benzo[*k*]fluoranthene (10 μM) in DMF.

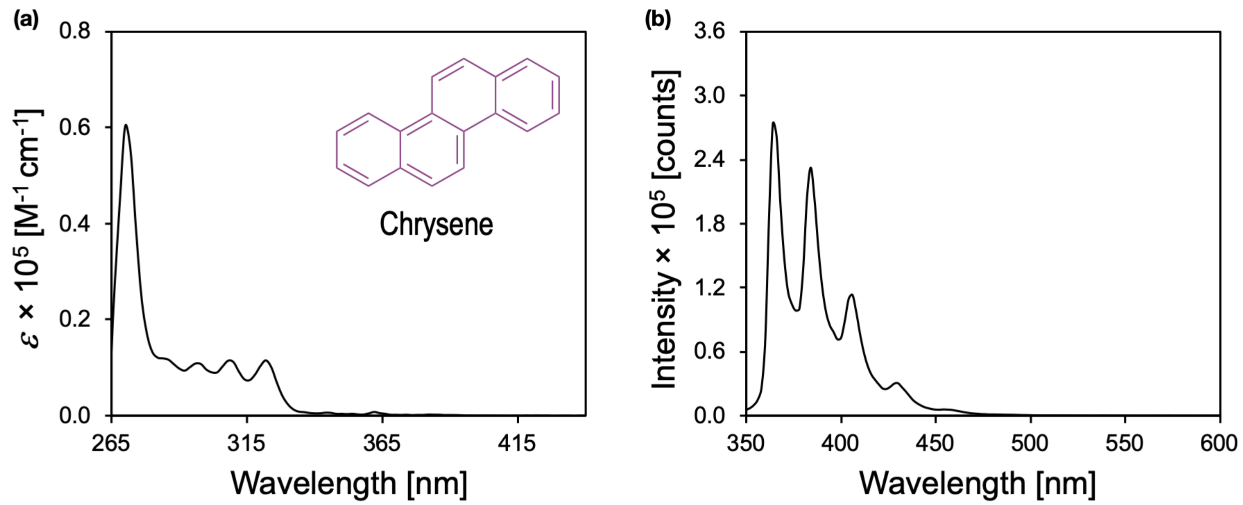

Fig. S10 (a) Molar extinction and (b) fluorescence (λ_{exc} = 330 nm) spectra for chrysene (10 μM) in DMF.

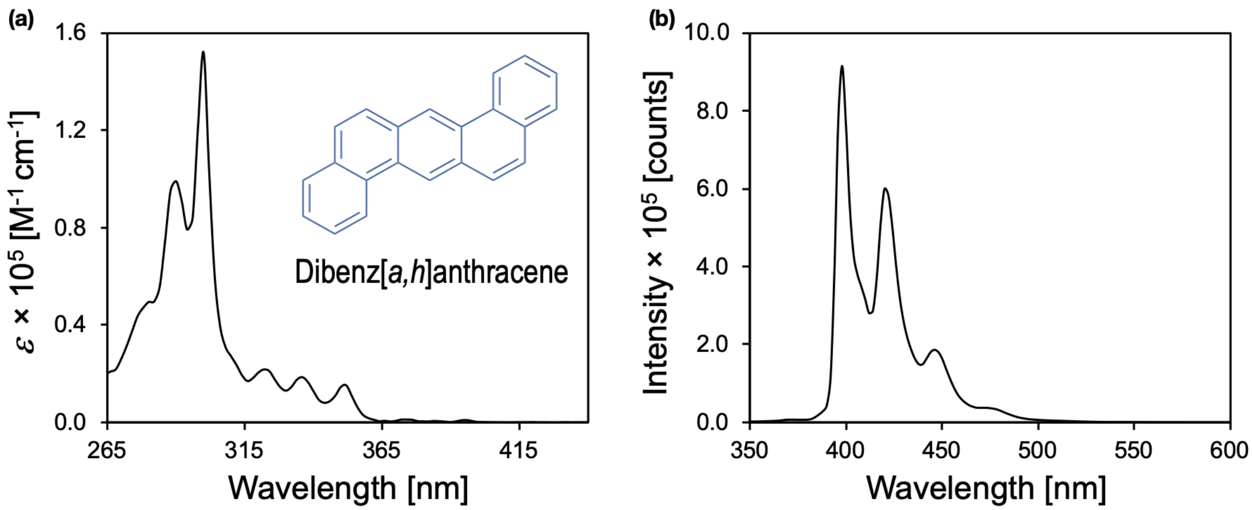

Fig. S11 (a) Molar extinction and (b) fluorescence (λ_{exc} = 330 nm) spectra for dibenz[*a,h*]anthracene (10 μM) in DMF.

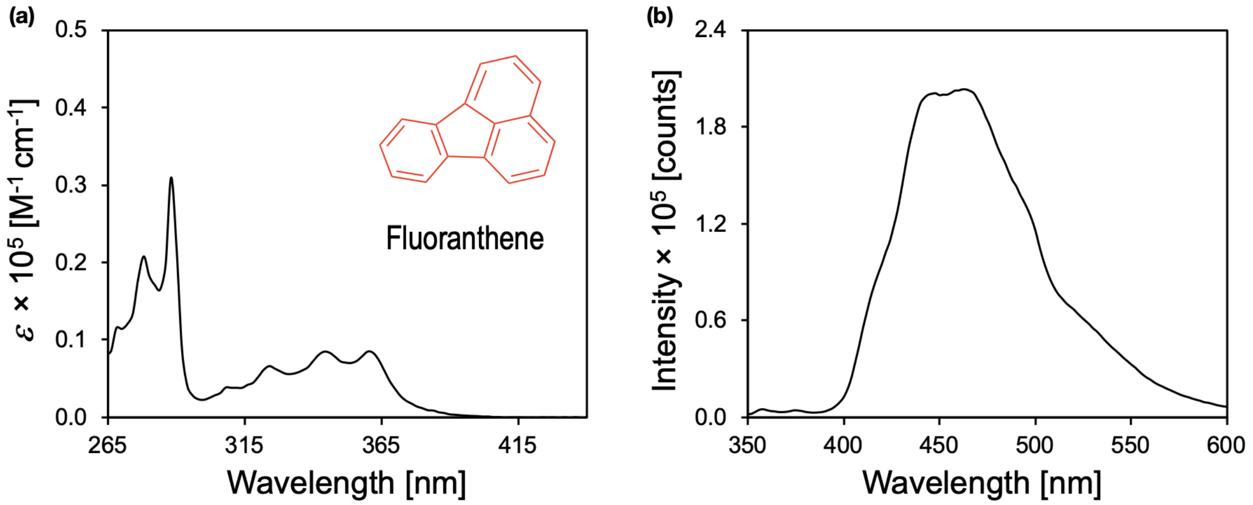

Fig. S12 (a) Molar extinction and (b) fluorescence (λ_{exc} = 330 nm) spectra for fluoranthene (10 μM) in DMF.

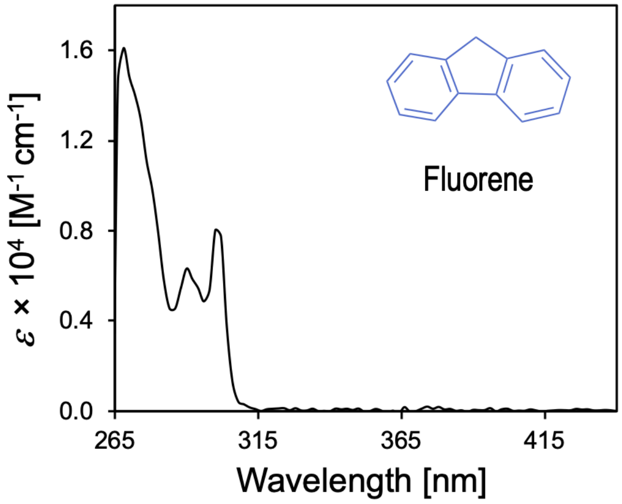

Fig. S13 Molar extinction spectra for fluorene (10 μM) in DMF. The fluorescence spectrum is not plotted as fluorene does not significantly absorb at λ_{exc} = 330 nm.

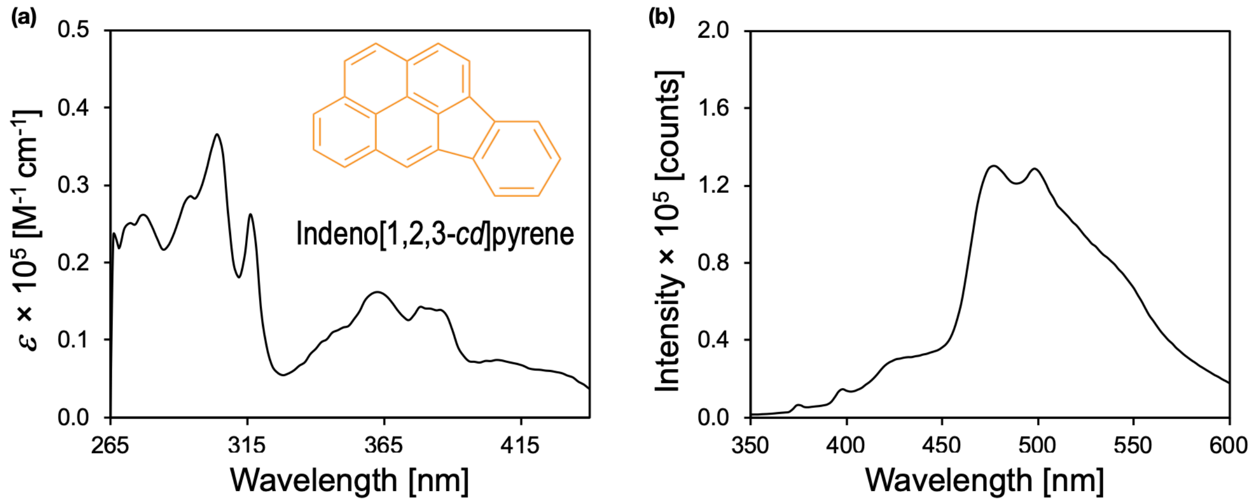

Fig. S14 (a) Molar extinction and (b) fluorescence (λ_{exc} = 330 nm) spectra for indeno[1,2,3-*cd*]pyrene (10 μM) in DMF.

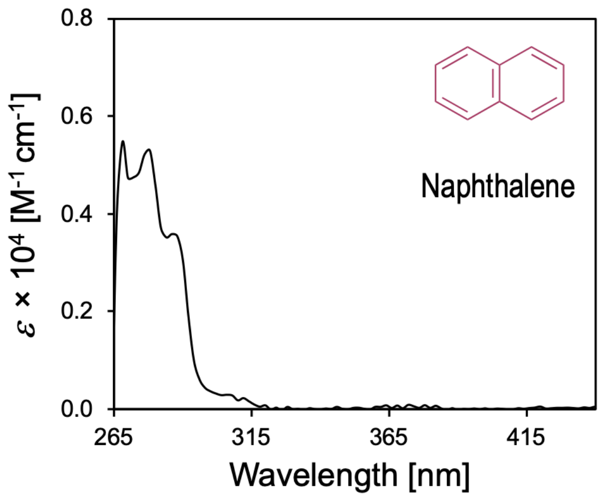

Fig. S15 Molar extinction spectra for naphthalene (10 μM) in DMF. The fluorescence spectrum is not plotted as naphthalene does not significantly absorb at λ_{exc} = 330 nm.

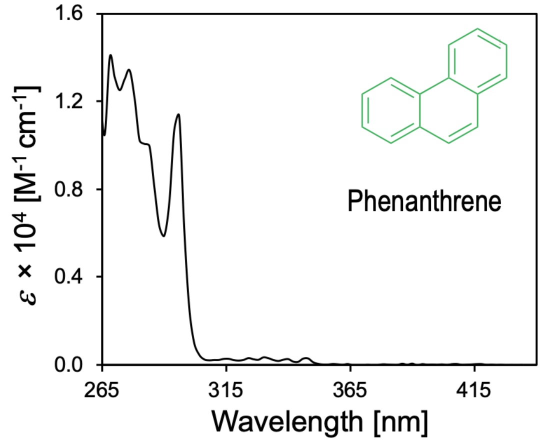

Fig. S16 Molar extinction spectra for phenanthrene (10 μM) in DMF. The fluorescence spectrum is not plotted as phenanthrene does not significantly absorb at λ_{exc} = 330 nm.

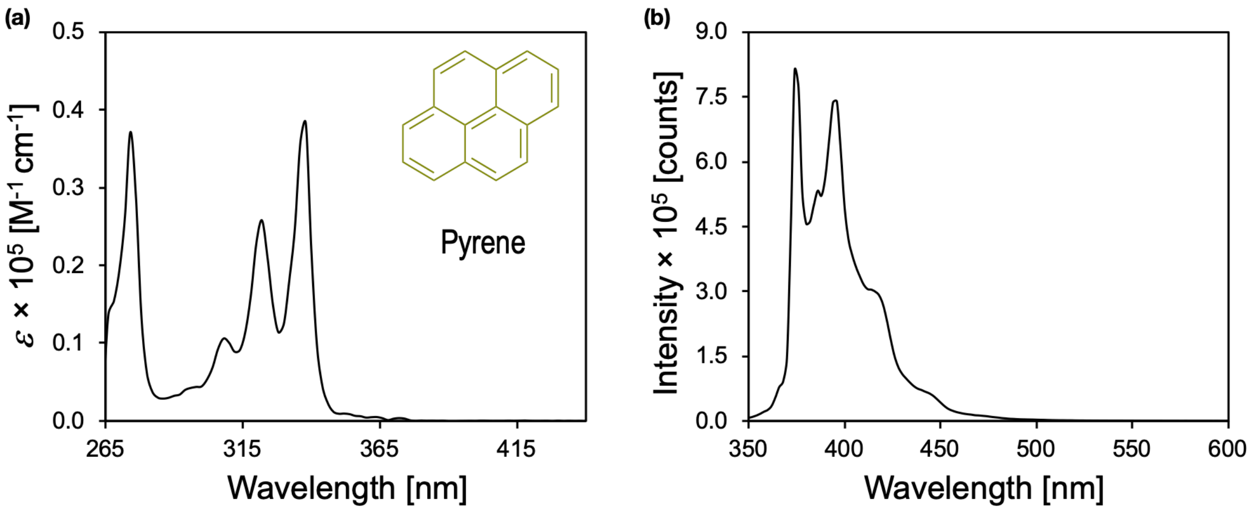

Fig. S17 (a) Molar extinction and (b) fluorescence (λ_{exc} = 330 nm) spectra for pyrene (10 μM) in DMF.

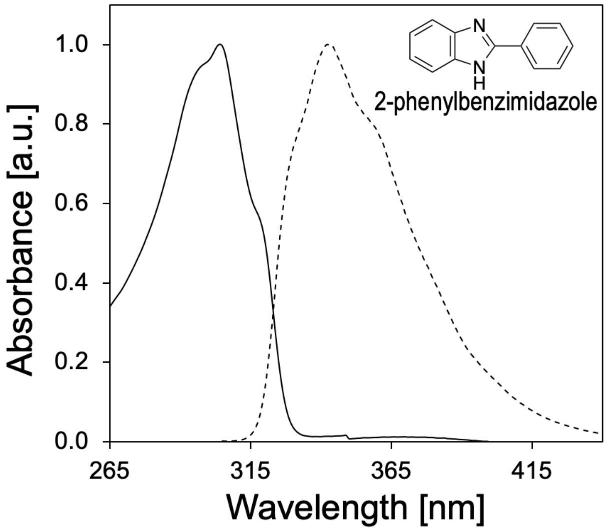

Fig. S18 Normalized absorbance (solid line) and fluorescence (dotted line) spectra of 2-phenylbenzimidazole (30 μM) in DMF.

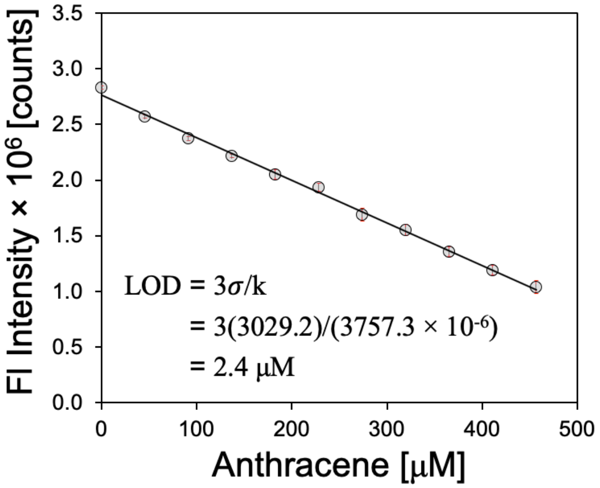

Fig. S19 Fluorescence intensity as anthracene is titrated into **P2** in DMF (15 mg L^{-1}). Titrations were performed in triplicate where fluorescence was collected at (λ_{exc} = 374 nm). The limit of detection was evaluated from the equation LOD = 3σ/k, where σ is the standard deviation in the emission intensity for the polymer solution without anthracene, and k is the slope of the calibration curve. Red error bars represent σ at each concentration.

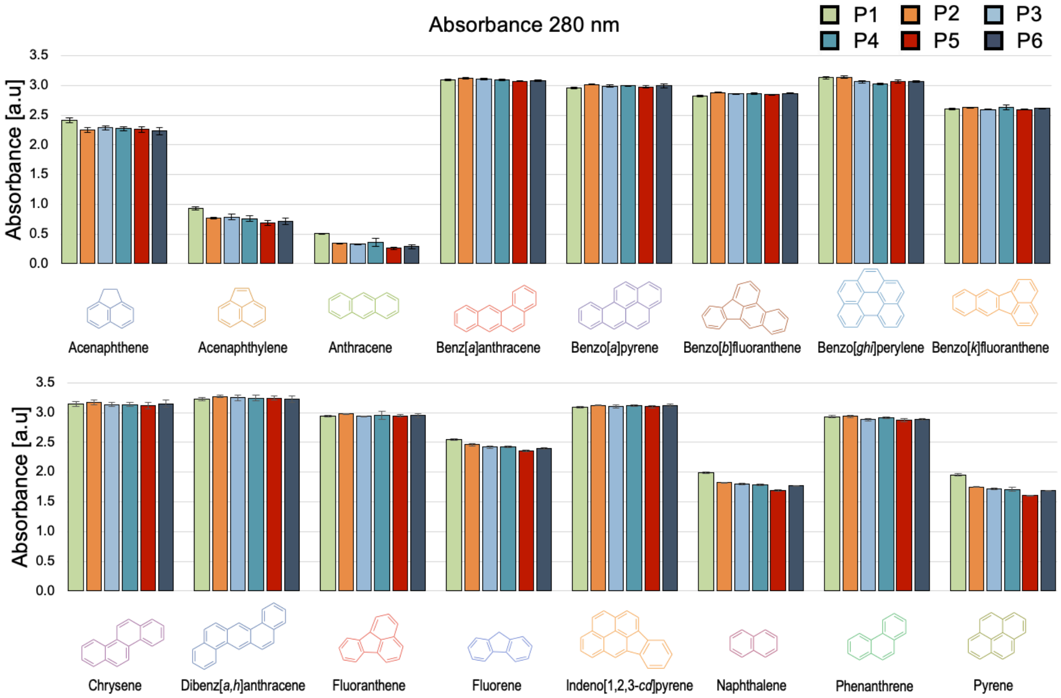

Fig. S20 Experimental absorbance at 280 nm of each polymer (15 mg L^{-1}) in the presence of the indicated PAH (500 µM). The reported value is the average of 12 replicates.

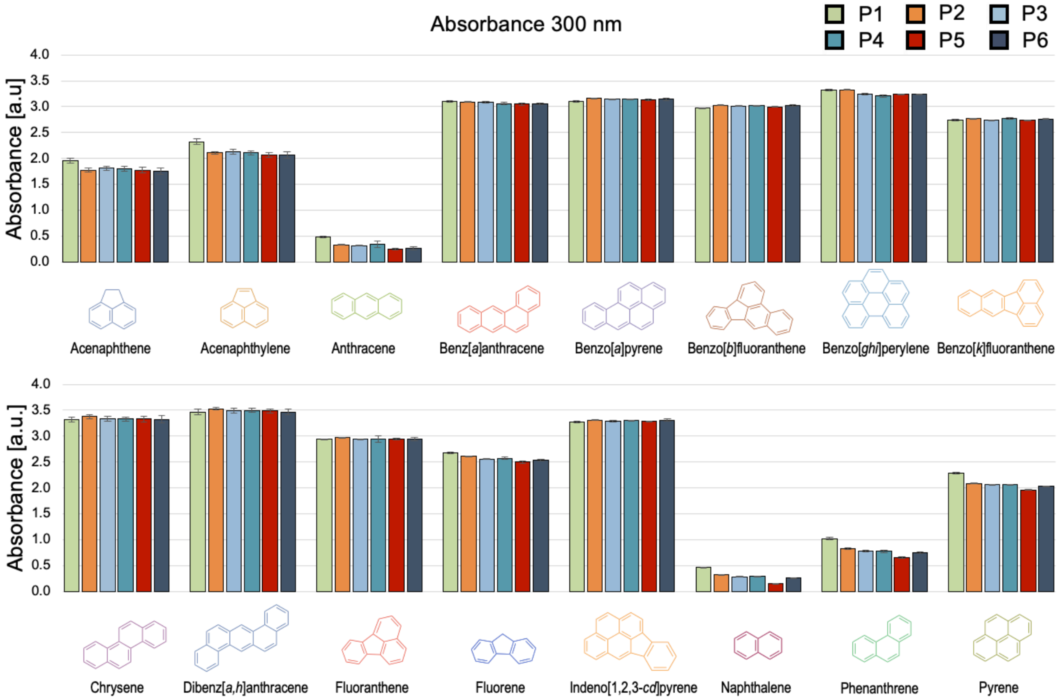

Fig. S21 Experimental absorbance at 300 nm of each polymer (15 mg L^{-1}) in the presence of the indicated PAH (500 µM). The reported value is the average of 12 replicates.

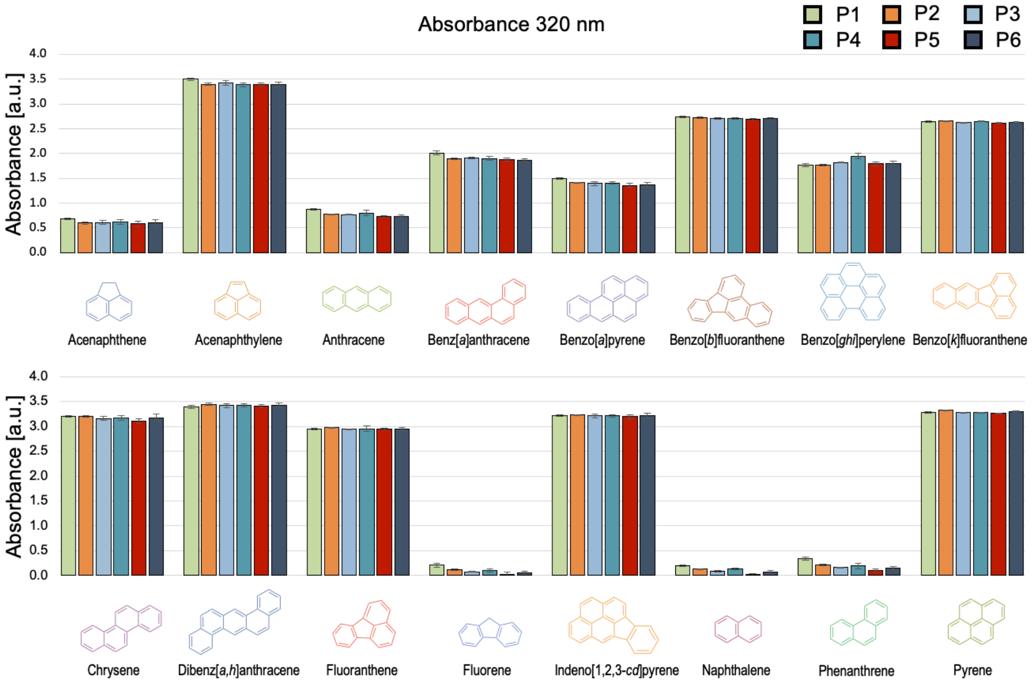

Fig. S22 Experimental absorbance at 320 nm of each polymer (15 mg L^{-1}) in the presence of the indicated PAH (500 µM). The reported value is the average of 12 replicates.

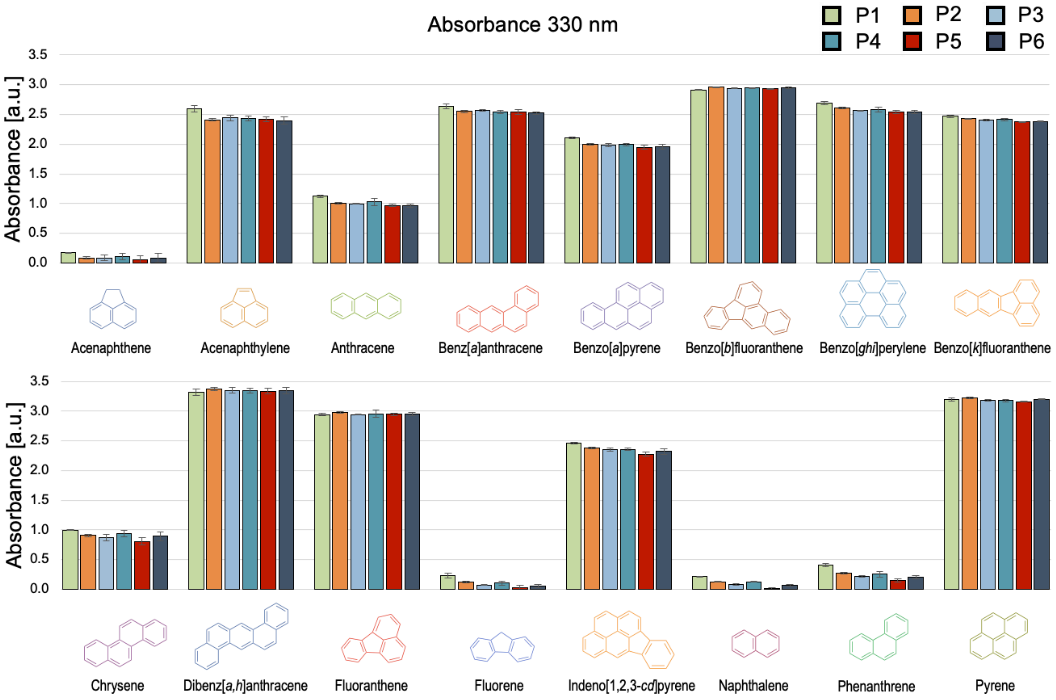

Fig. S23 Experimental absorbance at 330 nm of each polymer (15 mg L^{-1}) in the presence of the indicated PAH (500 µM). The reported value is the average of 12 replicates.

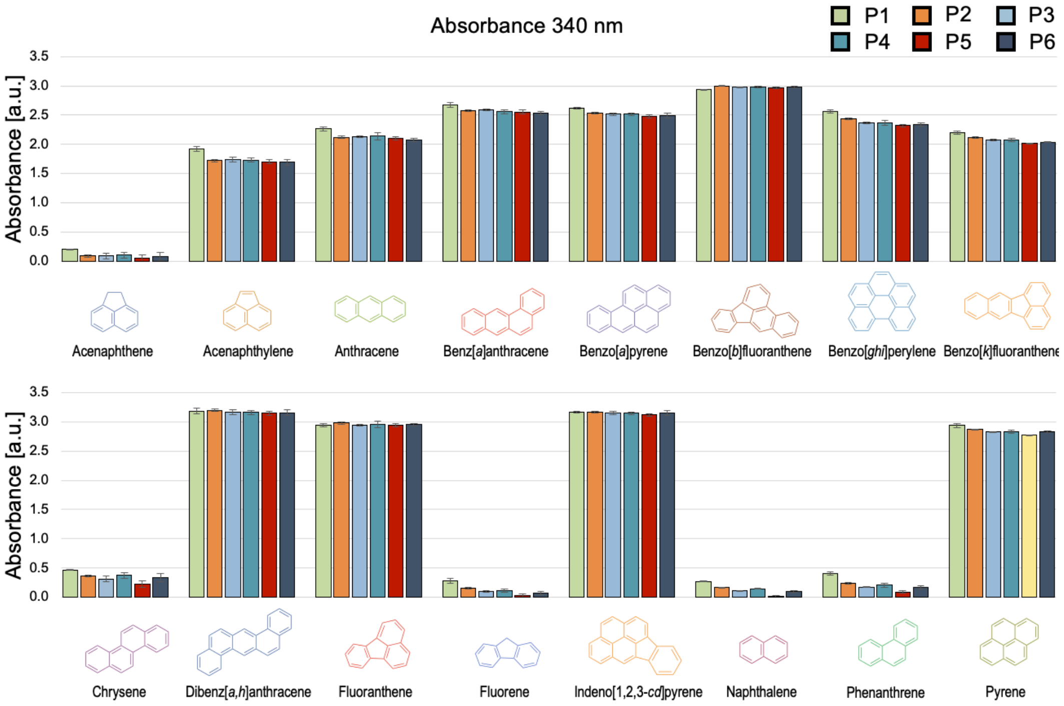

Fig. S24 Experimental absorbance at 340 nm of each polymer (15 mg L^{-1}) in the presence of the indicated PAH (500 μM). The reported value is the average of 12 replicates.

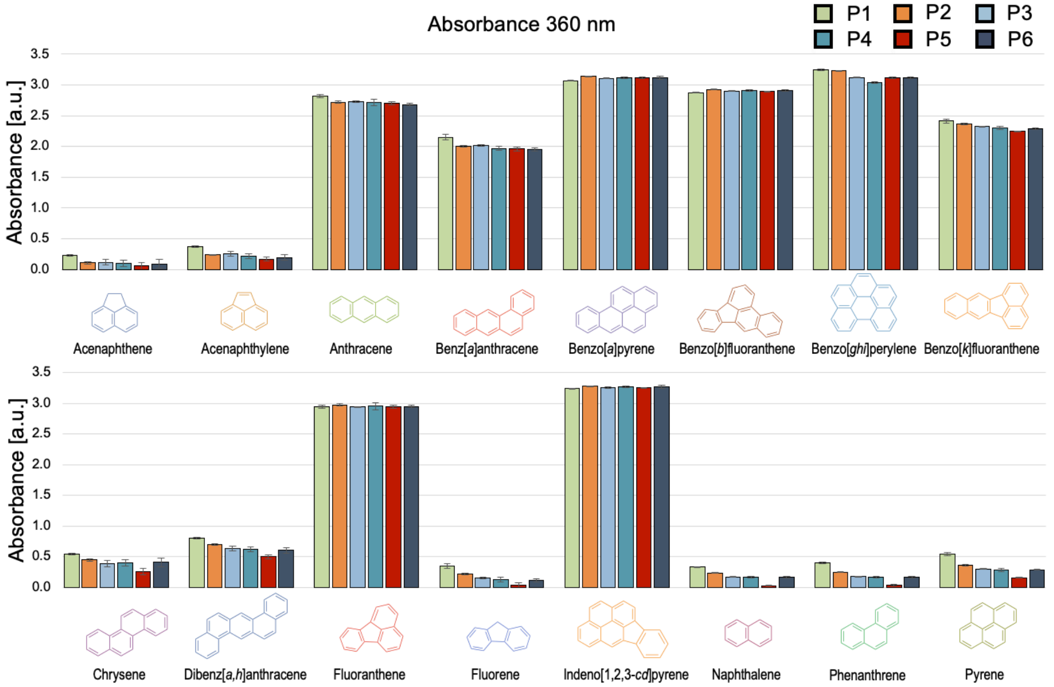

Fig. S25 Experimental absorbance at 360 nm of each polymer (15 mg L^{-1}) in the presence of the indicated PAH (500 μM). The reported value is the average of 12 replicates.

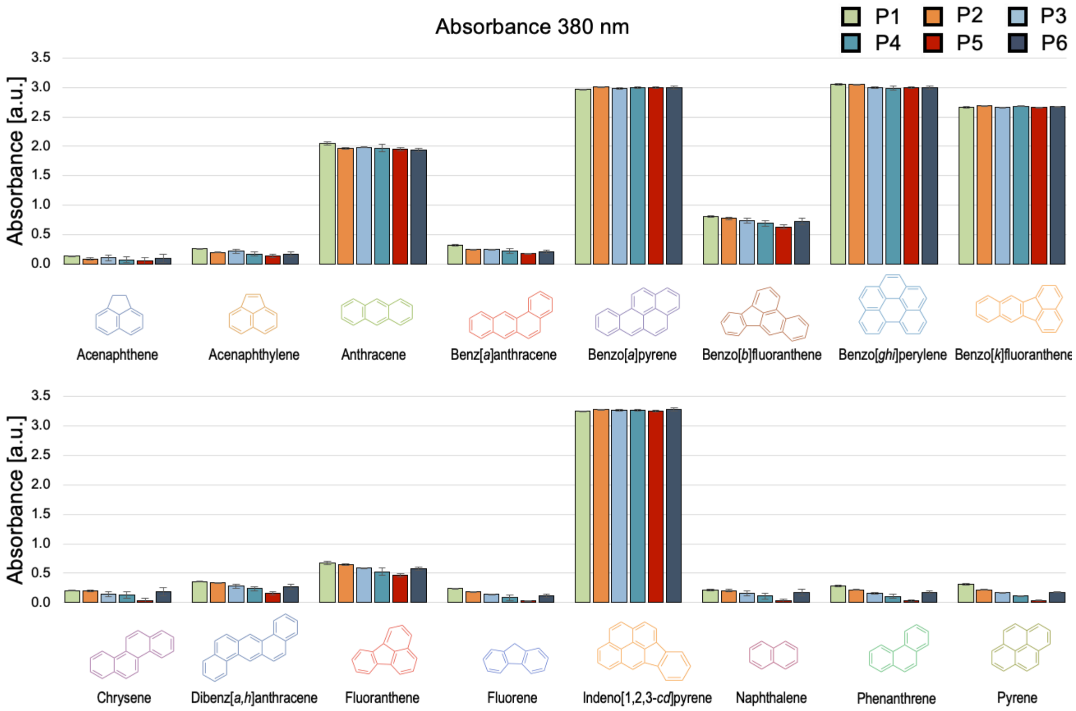

Fig. S26 Experimental absorbance at 380 nm of each polymer (15 mg L^{-1}) in the presence of the indicated PAH (500 µM). The reported value is the average of 12 replicates.

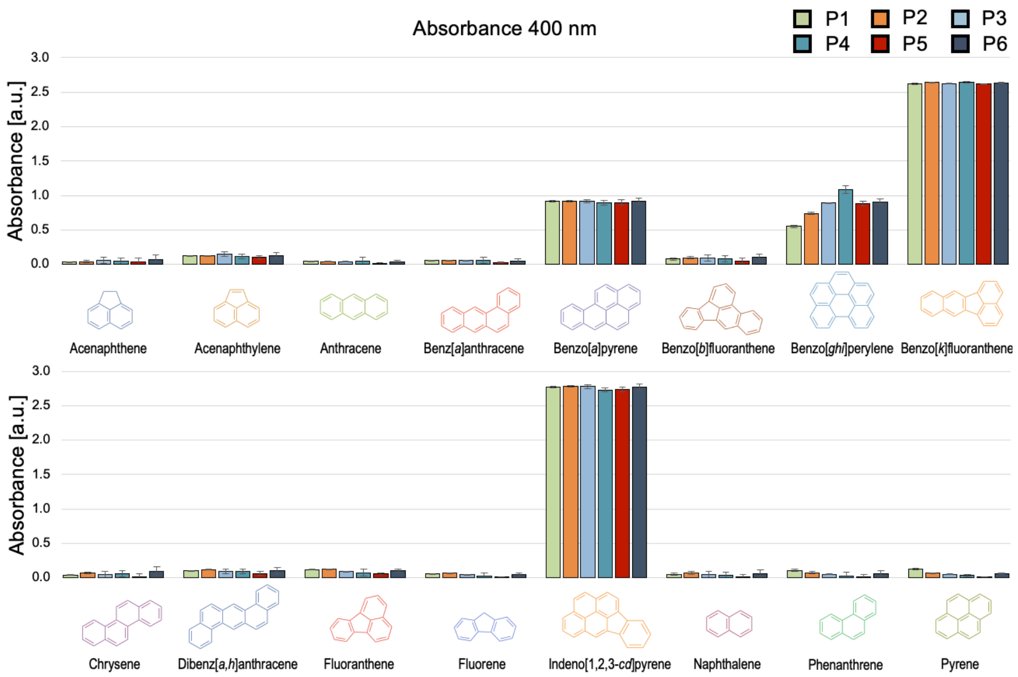

Fig. S27 Experimental absorbance at 400 nm of each polymer (15 mg L^{-1}) in the presence of the indicated PAH (500 µM). The reported value is the average of 12 replicates.

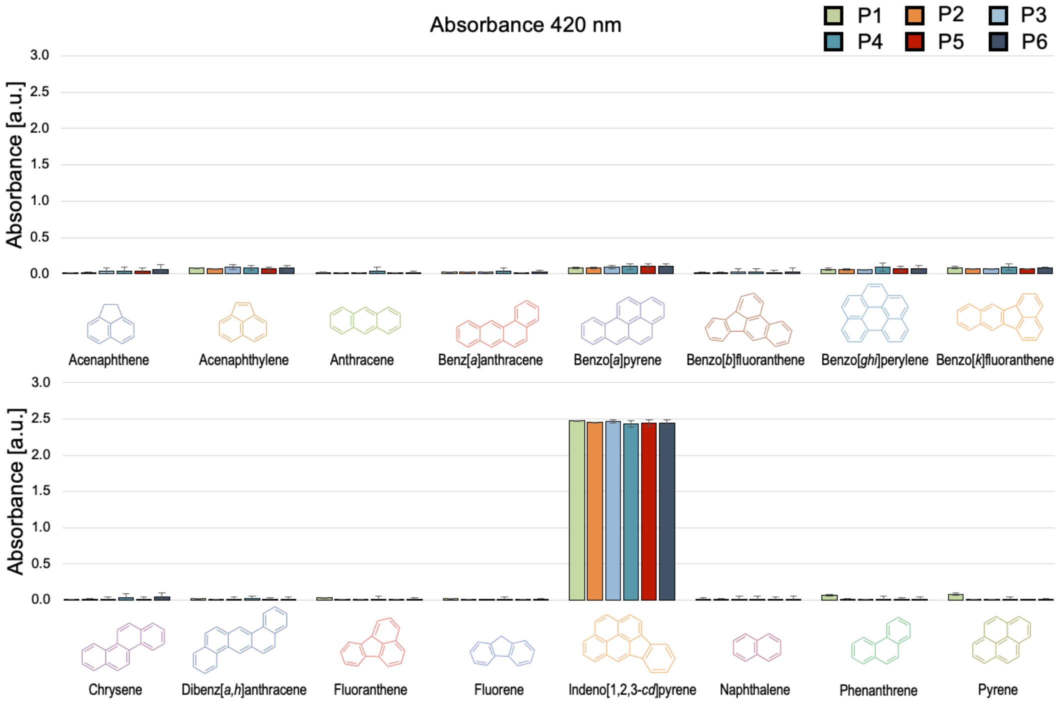

Fig. S28 Experimental absorbance at 420 nm of each polymer (15 mg L^{-1}) in the presence of the indicated PAH (500 µM). The reported value is the average of 12 replicates.

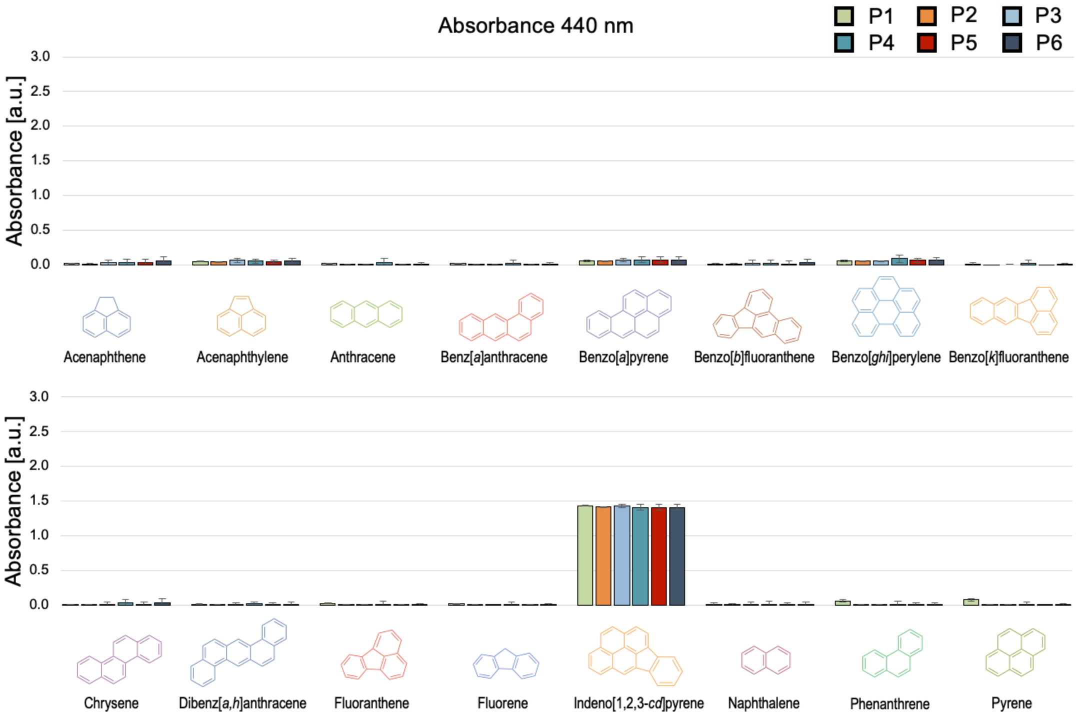

Fig. S29 Experimental absorbance at 440 nm of each polymer (15 mg L^{-1}) in the presence of the indicated PAH (500 µM). The reported value is the average of 12 replicates.

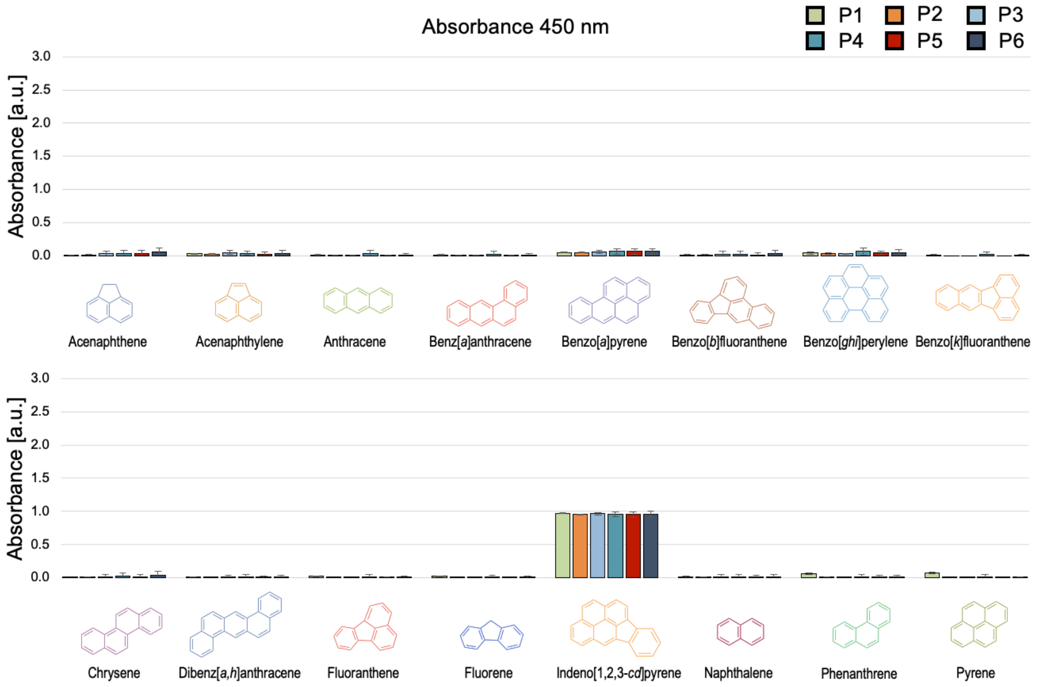

Fig. S30 Experimental absorbance at 450 nm of each polymer (15 mg L^{-1}) in the presence of the indicated PAH (500 μM). The reported value is the average of 12 replicates.

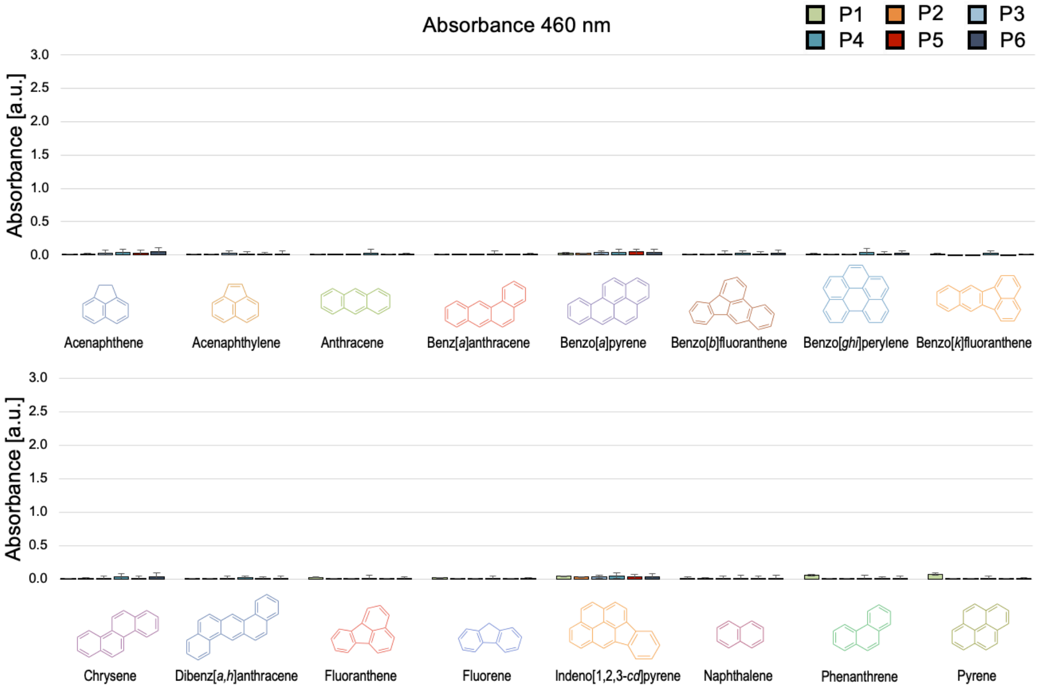

Fig. S31 Experimental absorbance at 460 nm of each polymer (15 mg L^{-1}) in the presence of the indicated PAH (500 μM). The reported value is the average of 12 replicates.

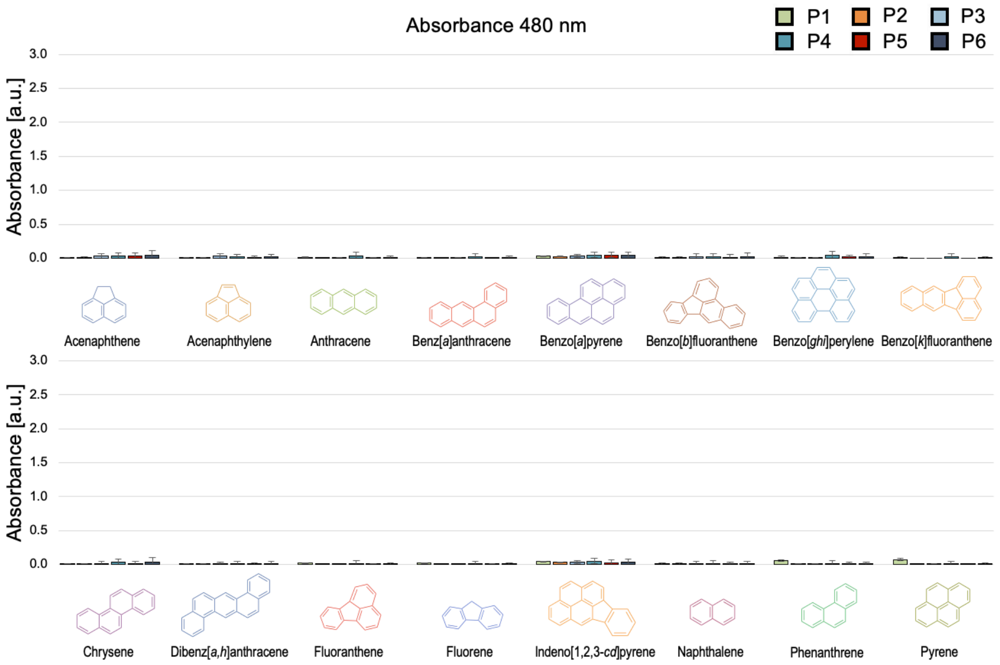

Fig. S32 Experimental absorbance at 480 nm of each polymer (15 mg L^{-1}) in the presence of the indicated PAH (500 µM). The reported value is the average of 12 replicates.

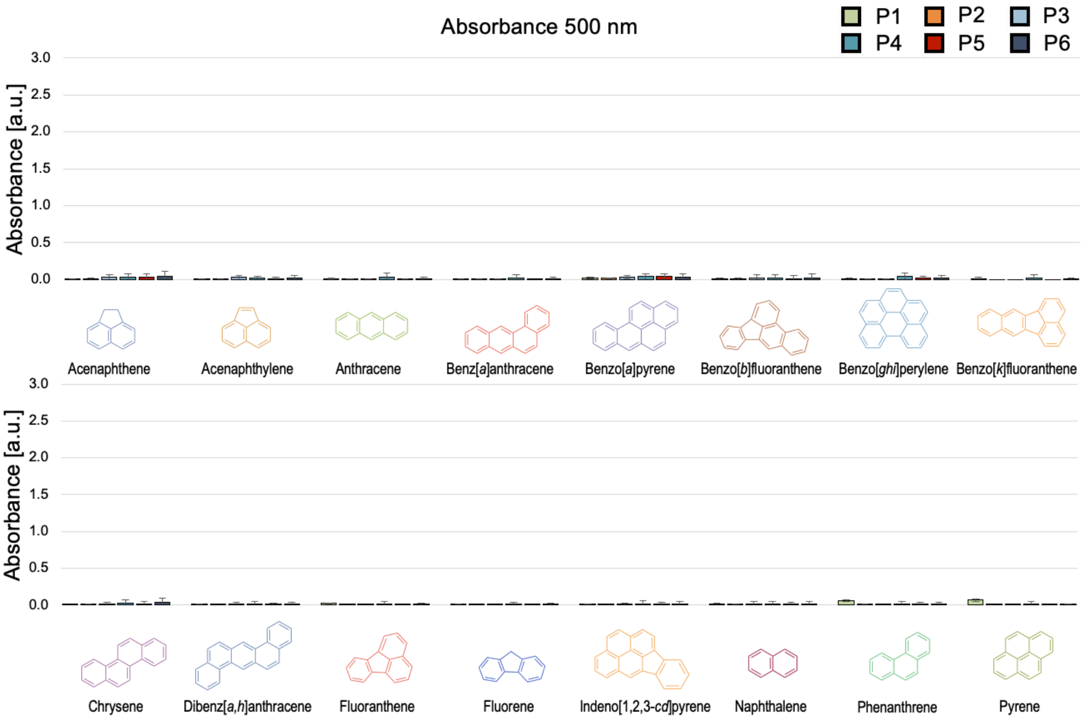

Fig. S33 Experimental absorbance at 500 nm of each polymer (15 mg L^{-1}) in the presence of the indicated PAH (500 µM). The reported value is the average of 12 replicates.

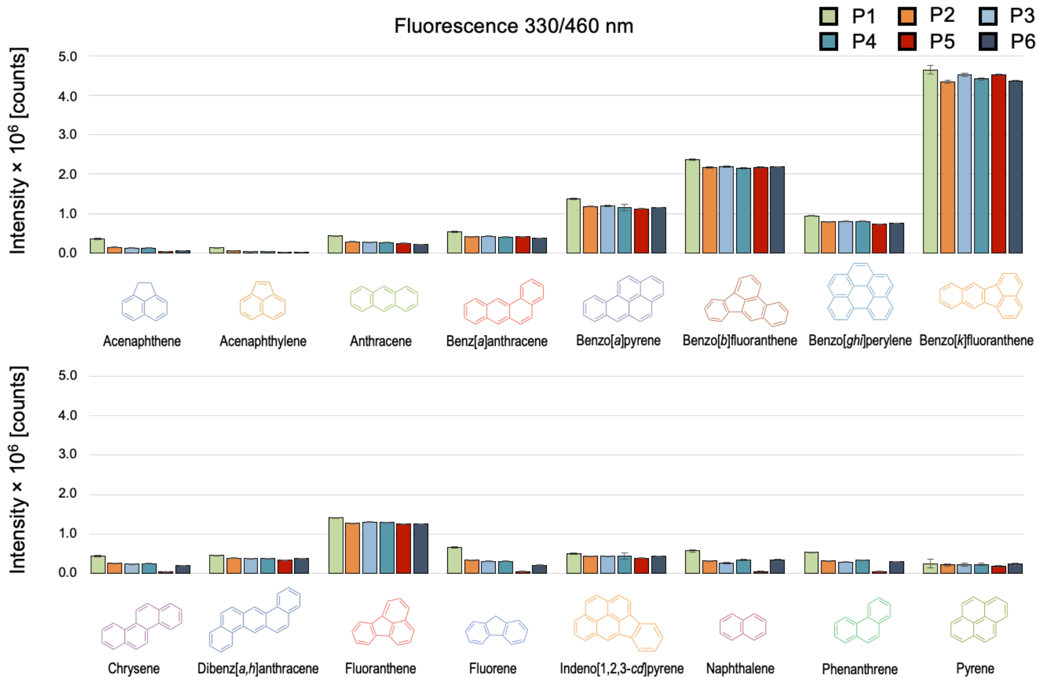

Fig. S34 Experimental fluorescence at 460 nm after excitation at 330 nm of each polymer (15 mg L^{-1}) in the presence of the indicated PAH (500 µM). The reported value is the average of 12 replicates.

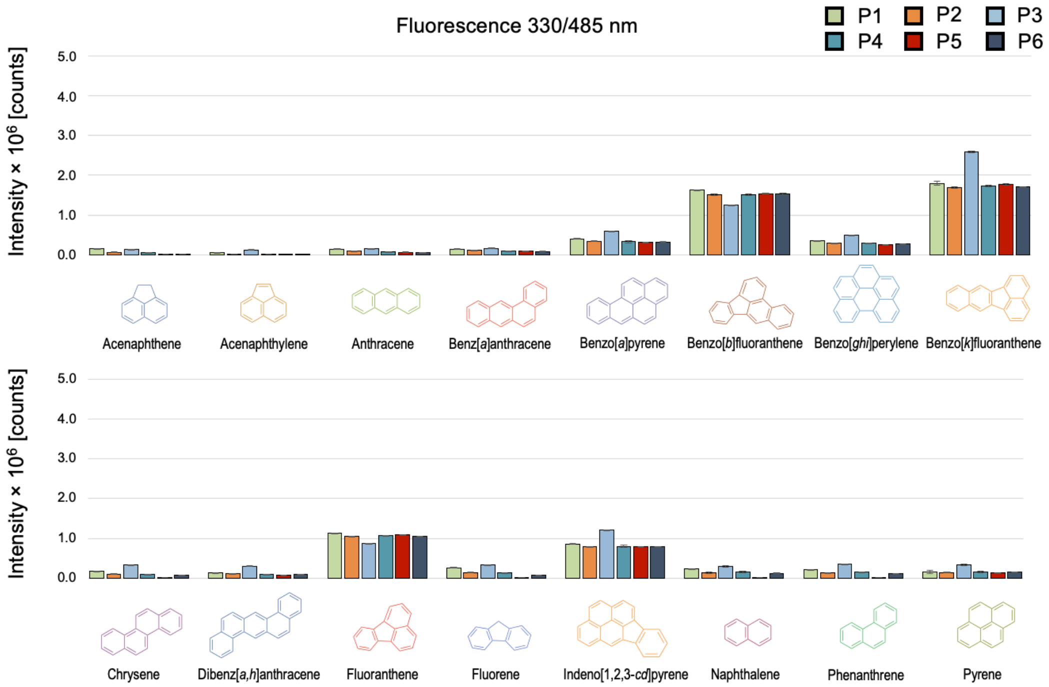

Fig. S35 Experimental fluorescence at 485 nm after excitation at 330 nm of each polymer (15 mg L^{-1}) in the presence of the indicated PAH (500 µM). The reported value is the average of 12 replicates.

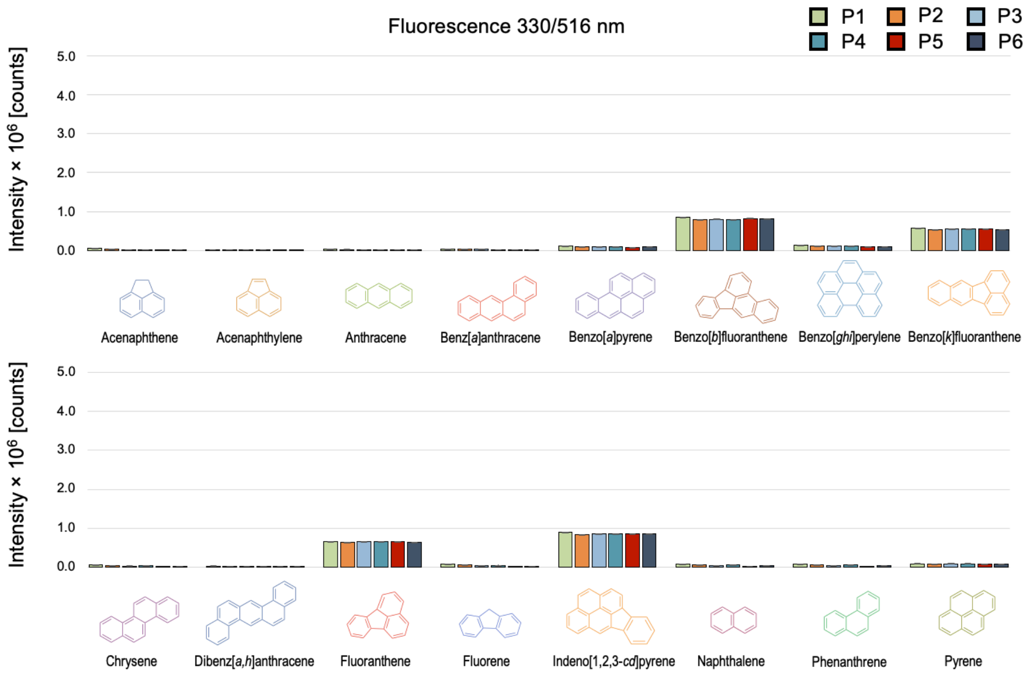

Fig. S36 Experimental fluorescence at 516 nm after excitation at 330 nm of each polymer (15 mg L^{-1}) in the presence of the indicated PAH (500 μM). The reported value is the average of 12 replicates.

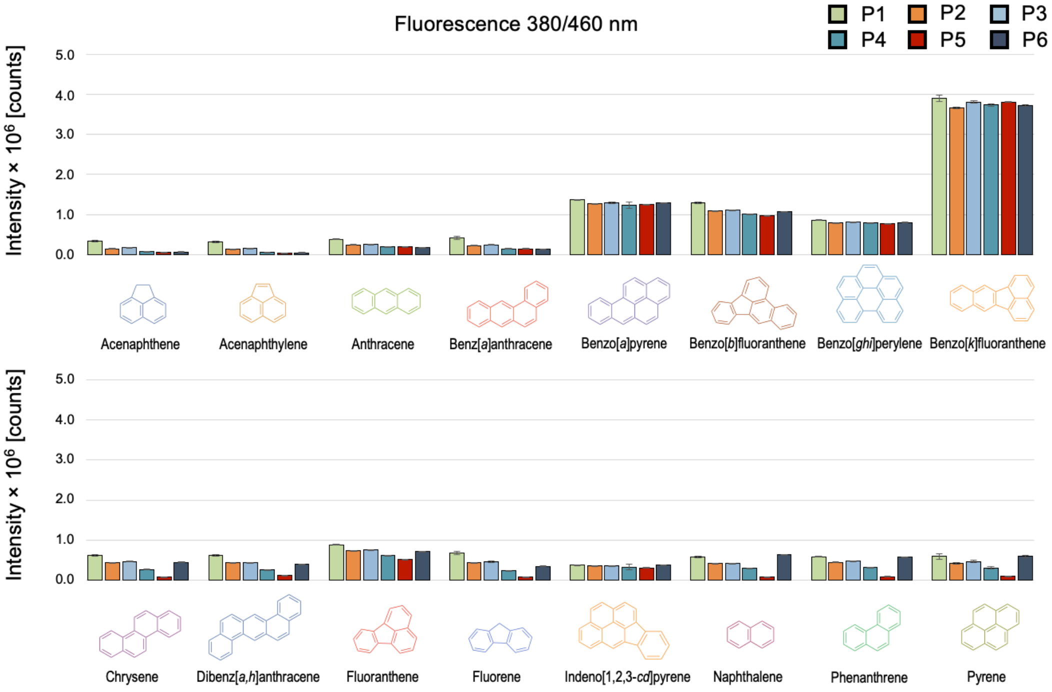

Fig. S37 Experimental fluorescence at 460 nm after excitation at 380 nm of each polymer (15 mg L^{-1}) in the presence of the indicated PAH (500 μM). The reported value is the average of 12 replicates.

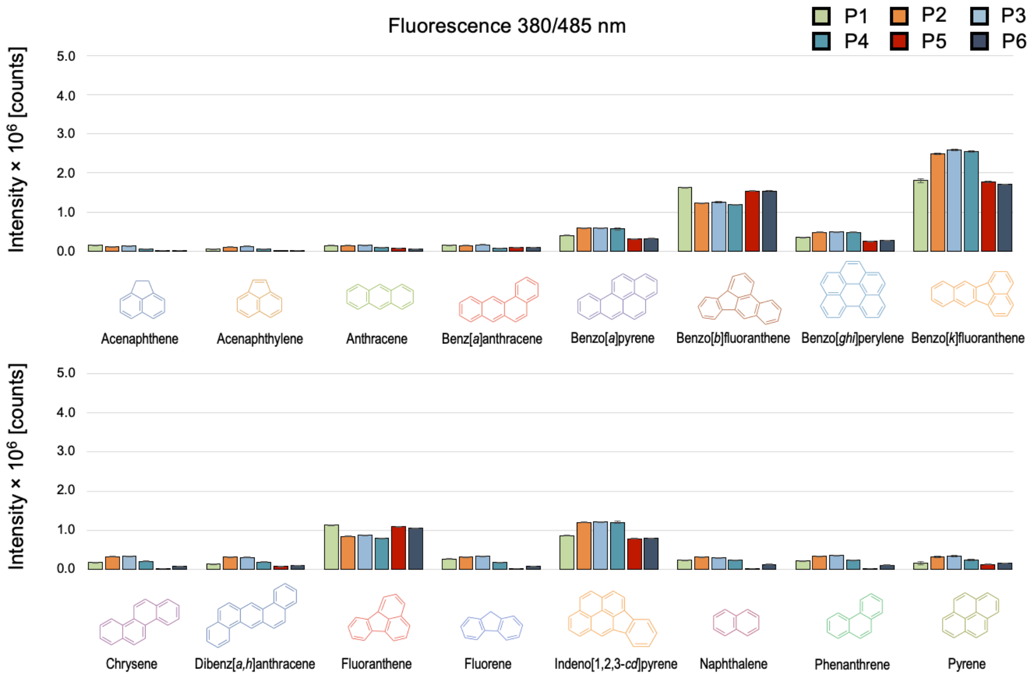

Fig. S38 Experimental fluorescence at 485 nm after excitation at 380 nm of each polymer (15 mg L^{-1}) in the presence of the indicated PAH (500 µM). The reported value is the average of 12 replicates.

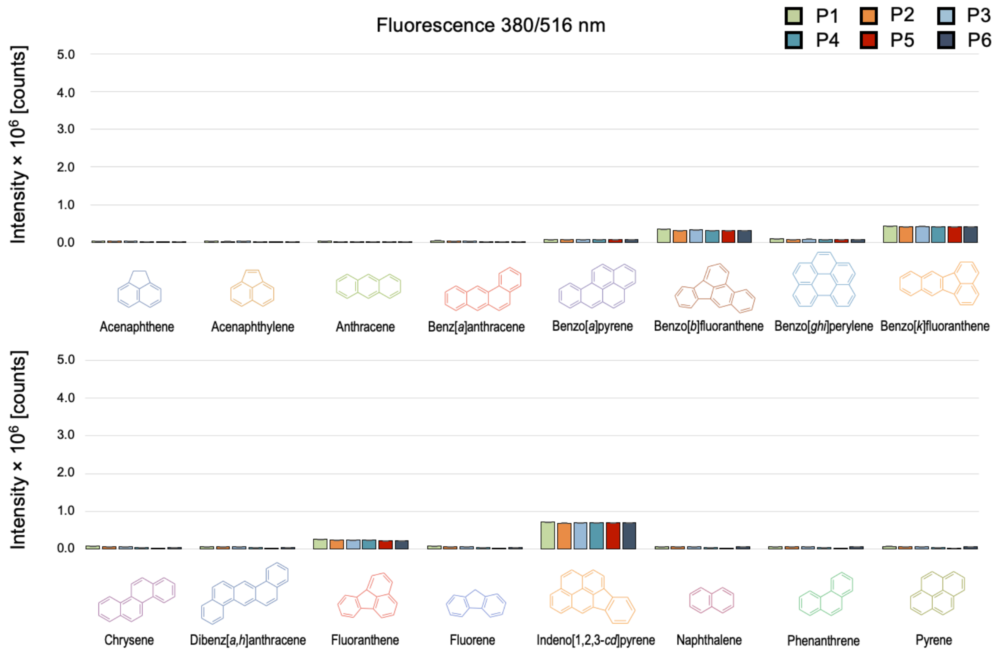

Fig. S39 Experimental fluorescence at 516 nm after excitation at 380 nm of each polymer (15 mg L^{-1}) in the presence of the indicated PAH (500 µM). The reported value is the average of 12 replicates.

3. Multivariate data analysis

In order to evaluate the discriminatory power of the molecular probes, Linear Discriminant Analysis (LDA) and Principal Component Analysis (PCA) were used to interpret the data sets. LDA and PCA are statistical treatments that are used for the interpretation of a multidimensional dataset. All multivariate analyses were performed in the commercial *Mathematica* program (release 11.3) published by Wolfram Research Inc.

As a first step towards data analysis, each variable in the raw experimental data set was mean-centered and standardized, a common practice in multivariate data analysis in the case of input data spanning very different dynamic ranges, as is the case in e.g. absorbance and fluorescence intensity raw data in our set. Shown below is the standardized instrumental response pattern for each instrumental measurement, presented as a heat map (**Fig. S40**).

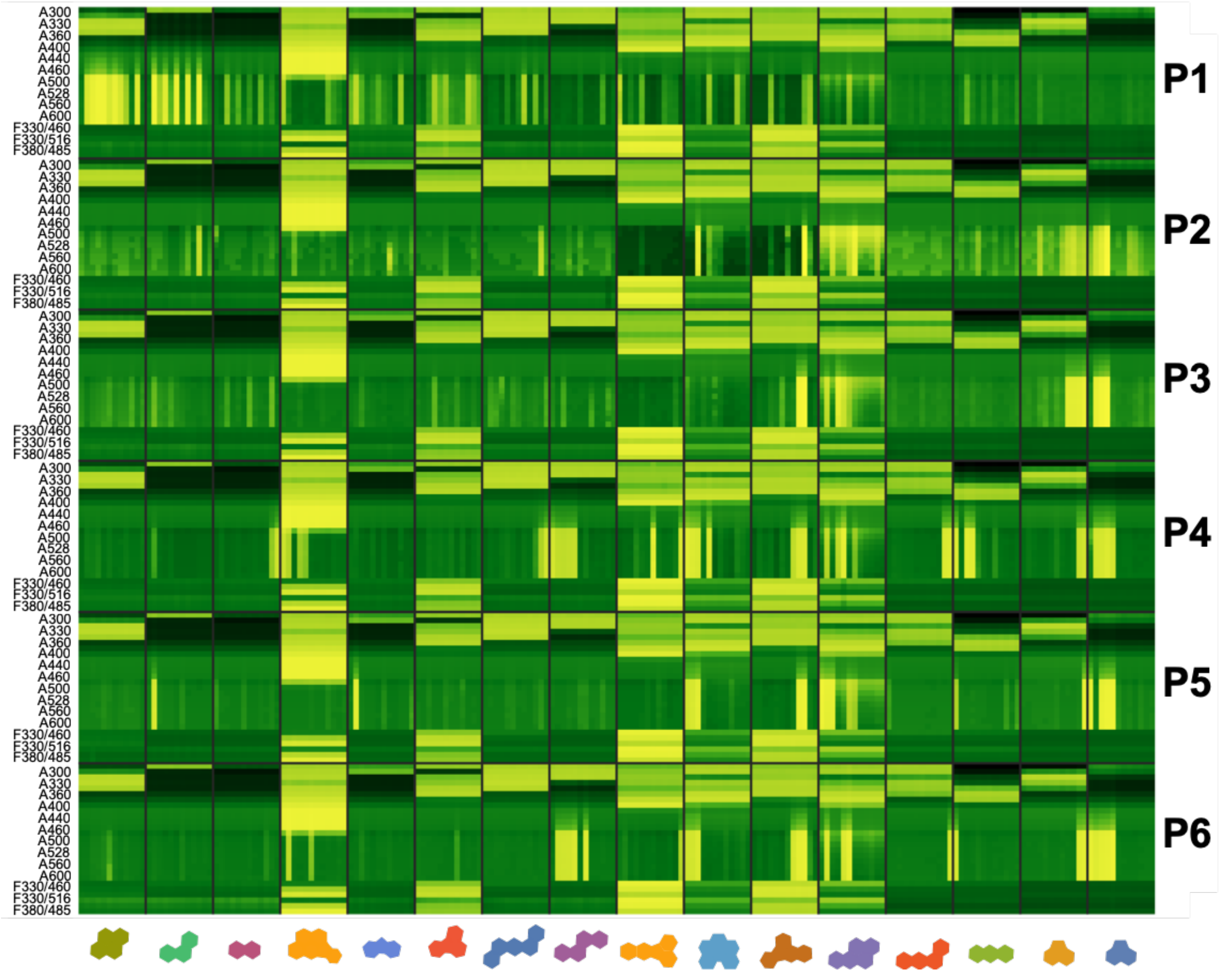

Fig. S40 A visual map of the experimental response to the target PAHs. "A" refers to an absorbance measurement, "F" to fluorescence emission.

Principal component analysis (PCA) was applied to each set of PAH/polymer replicate points. Points well outside a 95% confidence interval (CI) were considered outliers and removed from the raw data set. As an example, see **Fig. S41** for the pyrene analyte: in this case, data point 12 lies well outside the 95% CI and was removed from the data set before further analysis.

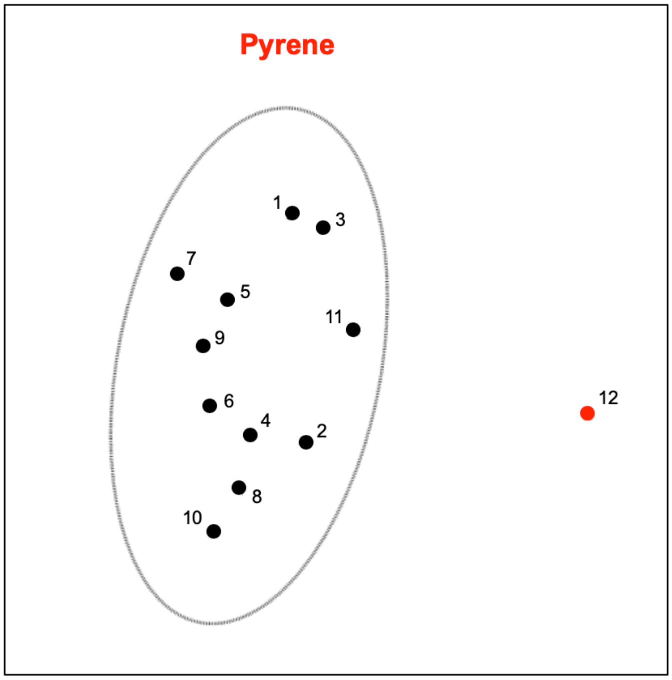

Fig. S41 PCA score plot of the pyrene replicate set, with 95% confidence ellipsoid shown as a dashed line. Point 12 (indicated in red), is clearly outside the confidence interval, and it was removed from the data set before further analysis.

In order to determine those instrumental measurements that do not contribute to the discriminatory power of the array and may even diminish it by introducing unwanted experimental noise, each instrumental variable was analyzed to provide an overview of the quality of information contained in each instrumental measurement. A visual representation of this analysis is presented in **Fig. S42** for the dataset that generated the scores plot in **Fig. 4a**. For each polymer, the absorbance measurements above 400 nm contribute more experimental noise than information, and therefore, these measurements were removed from the final dataset.

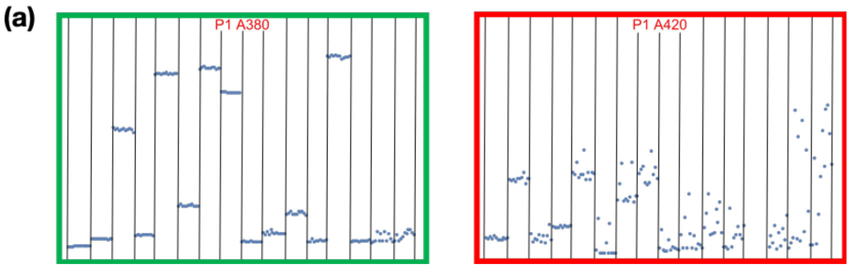

(b)

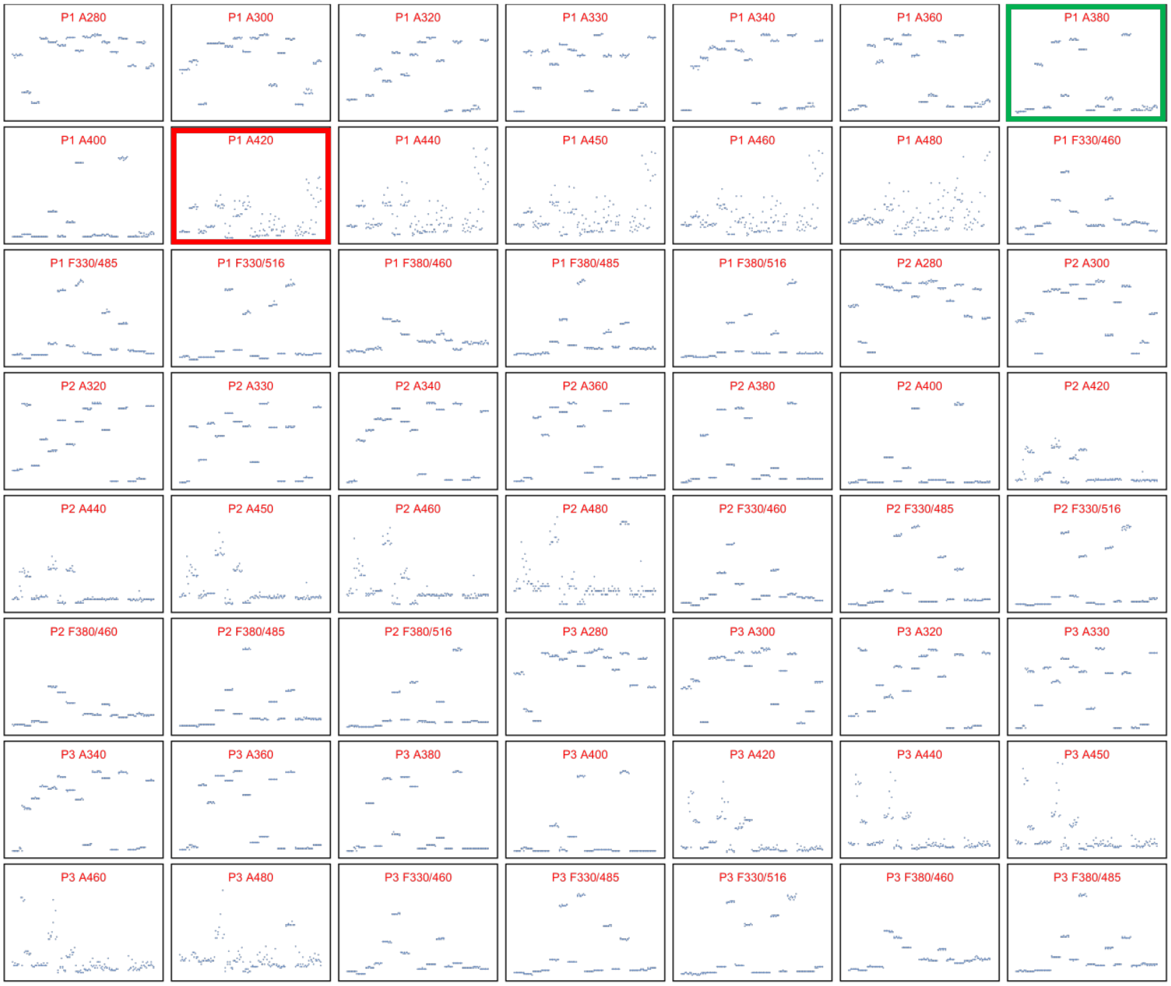
P1 A280
P1 A300
P1 A320
P1 A330
P1 A340
P1 A360
P1 A380
P1 A400
P1 A420
P1 A440
P1 A450
P1 A460
P1 A480
P1 F330/460
P1 F330/485
P1 F330/516
P1 F380/460
P1 F380/485
P1 F380/516
P2 A280
P2 A300
P2 A320
P2 A330
P2 A340
P2 A360
P2 A380
P2 A400
P2 A420
P2 A440
P2 A450
P2 A460
P2 A480
P2 F330/460
P2 F330/485
P2 F330/516
P2 F380/460
P2 F380/485
P2 F380/516
P3 A280
P3 A300
P3 A320
P3 A330
P3 A340
P3 A360
P3 A380
P3 A400
P3 A420
P3 A440
P3 A450
P3 A460
P3 A480
P3 F330/460
P3 F330/485
P3 F330/516
P3 F380/460
P3 F380/485

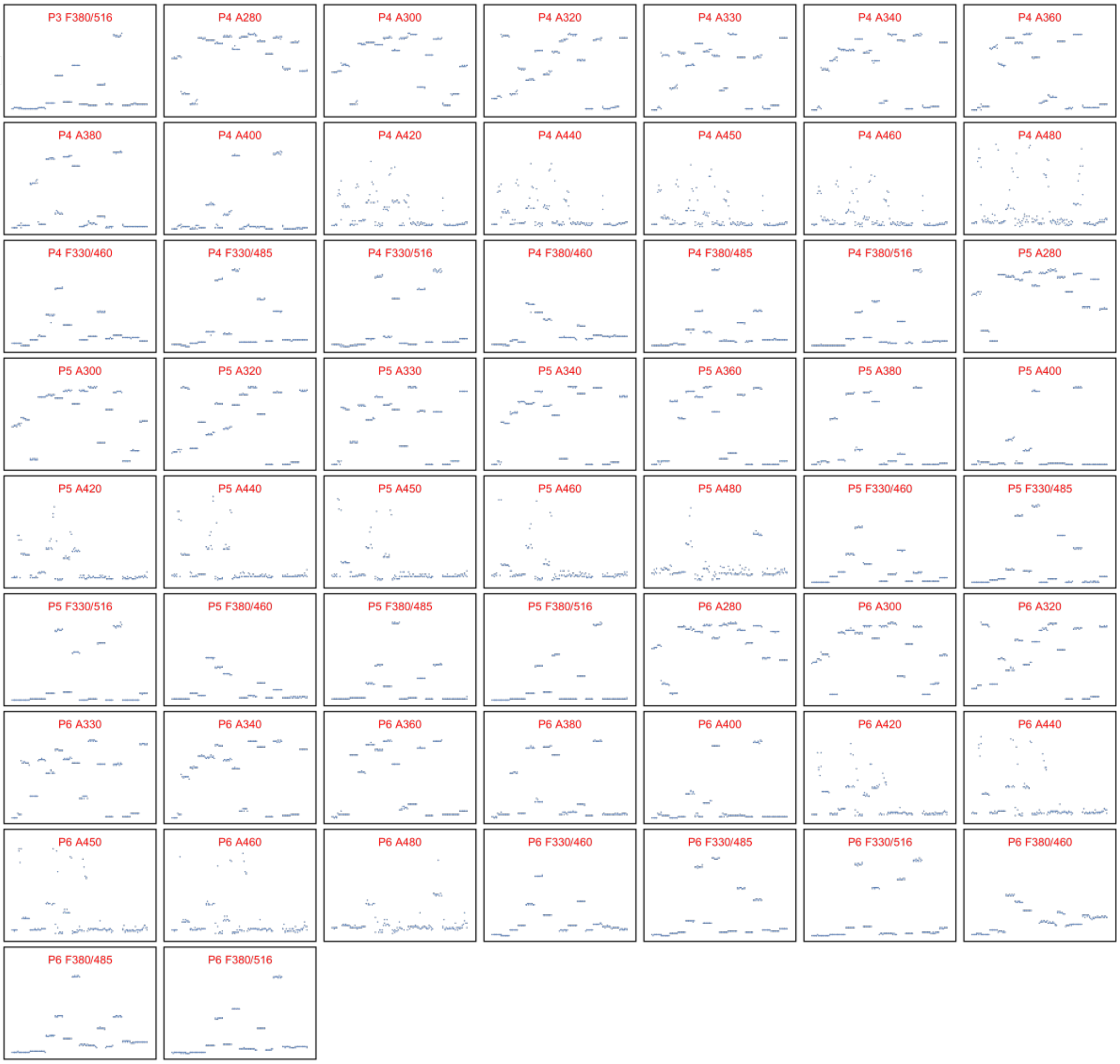

Fig. S42 (a) An example of one information rich (green) vs. an information poor (red) measurement for all 16 PAH analytes. (b) A visual overview of the information content of each instrumental measurement in the dataset from which the LDA scores plot shown in **Fig. 4a** was generated. "A" refers to an absorbance measurement and "F" to fluorescence emission. For instance, "P1 A380" indicates absorbance of **P1** at 380 nm, and "P1 F330/460" indicates fluorescence emission of **P1** at 330/460 ($\lambda_{exc}/\lambda_{em}$).

The resulting dataset containing the selected 79 instrumental measurements was subjected to LDA analysis. The scores plot in **Fig. 5a** retains about half of the total information in the first two factors that can be used to generate a two-dimensional plot. However, it is very likely that the third factor contains a significant portion of discriminatory information. By including this third factor, a three-dimensional plot could be generated, which would retain roughly three-quarters of the information from the original dataset. The three-dimensional scores plot is presented in **Fig. S43**.

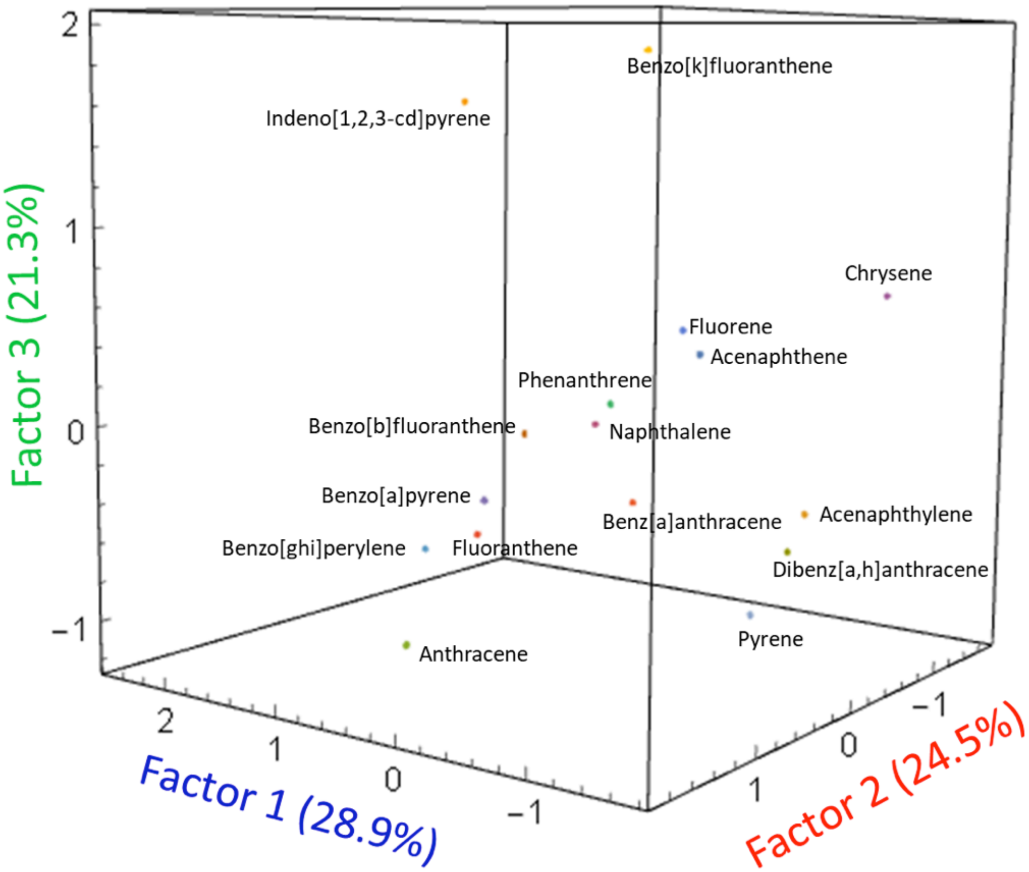

Fig. S43 The three-dimensional plot of the LDA scores for the differentiation of 16 PAHs with polymers **P1-P6**. This plot captures 74.7% of the total information contained in the raw dataset ([PAH] = 500 µM; [polymers] = 15 mg L^{-1}).

A similar method of multivariate data analysis is principal component analysis (PCA). This pattern-recognition technique is a useful partner to LDA because it provides a hypothesis-free representation of the discriminatory ability of the dataset. Shown below in **Fig. S44** are the two-dimensional

PCA plots for the datasets containing measurements taken with and without the use of the polymers. Similar to the LDA analysis, all 16 PAHs could be differentiated only when the polymers are used.

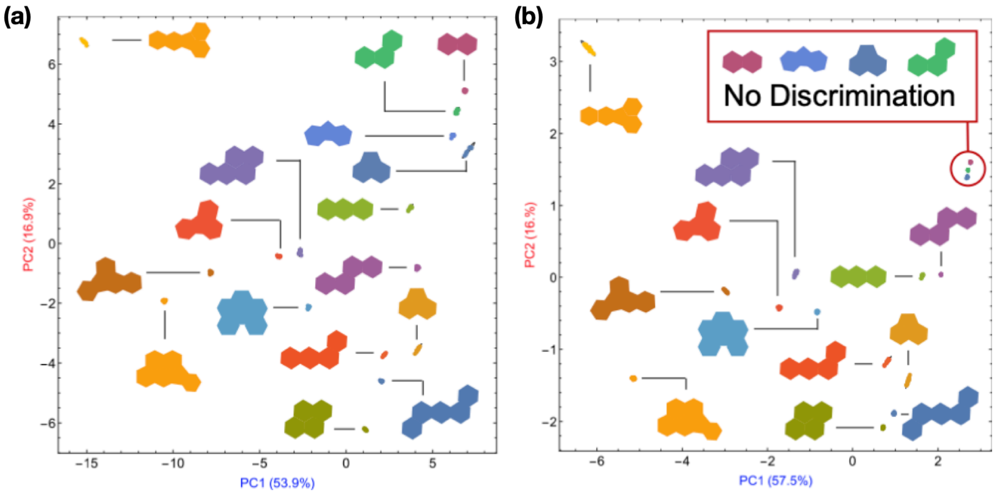

Fig. S44 PCA scores plot for the (a) differentiation of 16 PAHs using **P1**-**P6** and (b) the attempted differentiation of 16 PAHs without **P1**-**P6**.

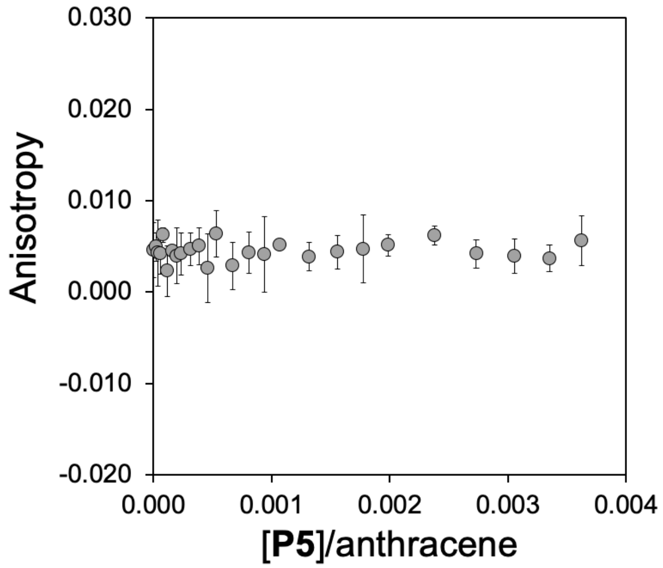

Fig. S45 Fluorescence anisotropy as **P5** is titrated into anthracene (100 μM) in DMF. If binding occurred between anthracene and **P5**, there should be a significant increase in the fluorescence anisotropy, indicating that there is lower rotational freedom for some of the anthracene molecules in solution.

4. Synthesis of functionalized monomers

2,7-dibromo-9,9-bis(10-bromodecyl)-9*H*-fluorene (SI-0)

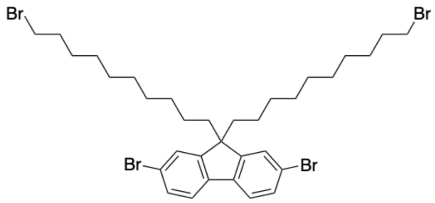

2,7-dibromofluorene (4.86 g, 15.0 mmol), 1,10-dibromodecane (9.00 g, 30.0 mmol), and tetrabutylammonium bromide (481 mg, 1.50 mmol) were dissolved in toluene (60 mL). 50% NaOH (30 mL) in water was added at room temperature and the mixture was heated to 60 °C for 12 h. The reaction mixture was then cooled to room temperature, and the aqueous layer was removed. The organic layer was then washed with water (3 × 50 mL) and dried over anhydrous $MgSO_4$. Volatiles were removed in vacuo, and purification was accomplished by flash chromatography using hexanes affording, 4.22 g of a colorless oil (5.54 mmol, 37%). Data are as follows: ^{1}H NMR (600 MHz, $CDCl_3$): δ 7.52 (d, *J* = 8.0 Hz, 2H), 7.46 (dd, *J* = 8.0, 1.8 Hz, 2H), 7.44 (d, *J* = 1.7 Hz, 2H), 3.38 (t, *J* = 6.9 Hz, 4H), 1.94–1.87 (m, 4H), 1.87–1.75 (m, 4H), 1.42–1.31 (m, 4H), 1.30–1.15 (m, 8H), 1.14–1.00 (m, 12H), 0.66–0.50 (br, 4H); ^{13}C NMR (151 MHz, $CDCl_3$): δ 152.67, 139.23, 130.32, 126.32, 121.62, 121.29, 55.83, 40.26, 34.15, 32.96, 29.91, 29.46, 29.41, 29.21, 28.81, 28.26, 23.72. HRMS (ESI) (*m/z*): $[M+H]^+$ calcd. for $C_{33}H_{47}Br_4$: 781.02252, found: 781.02163.

4.1 General Procedure A: Synthesis of Functionalized Monomers.

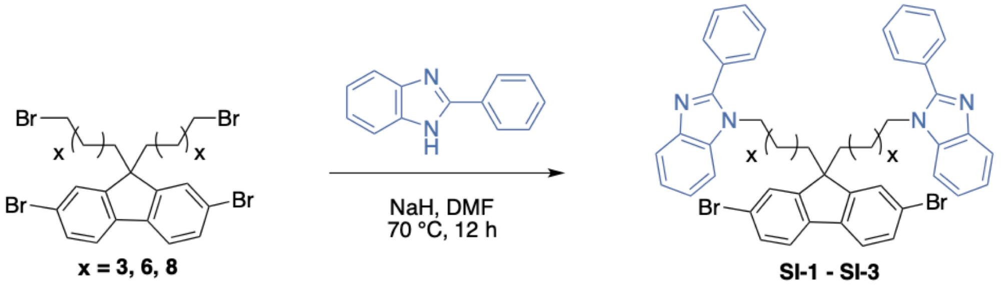

2,7-dibromo-9,9-bis(6-bromoalkyl)-9*H*-fluorene (1.02 mmol, 1.00 equiv.), 2-phenylbenzimidazole (2.24 mmol, 2.20 equiv.), NaH (4.08 mmol, 4.00 equiv.) were dissolved in DMF (10 mL) under nitrogen. The mixture was stirred at 70 °C for 12 h. The reaction mixture was then cooled to room temperature, quenched with deionized water, and extracted with ethyl acetate (3 × 50 mL). The organic layer was then washed

with water (3 × 50 mL) and dried over anhydrous $MgSO_4$. Volatiles were removed in vacuo, and purification was accomplished by flash chromatography using a hexanes:ethyl acetate gradient.

1,1'-((2,7-dibromo-9*H*-fluorene-9,9-diyl)bis(pentane-5,1-diyl))bis(2-phenyl-1*H*-benzo[*d*]imidazole) (SI-1)

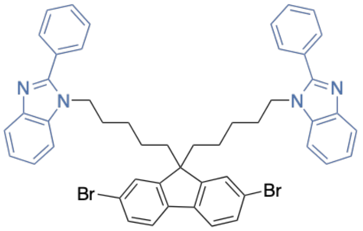

Compound **SI-1** was prepared following General Procedure A, using 2,7-dibromo-9,9-bis(5-bromopentyl)-9*H*-fluorene[1] (635 mg, 1.02 mmol), 2-phenylbenzimidazole (435 mg, 2.24 mmol), and NaH (97.9 mg, 4.08 mmol). Purification was accomplished by flash chromatography using a hexanes:ethyl acetate gradient, affording 581 mg (0.685 mmol, 67%) of a white solid. Data are as follows: ^{1}H NMR (600 MHz, $CDCl_3$): δ 7.82–7.78 (m, 2H), 7.63–7.60 (m, 4H), 7.50–7.43 (m, 10H), 7.32 (d, J = 1.7 Hz, 2H), 7.30–7.27 (m, 6H), 4.06 (t, J = 7.5 Hz, 4H), 1.78–1.75 (m, 4H), 1.57–1.52 (m, 4H), 1.00–0.95 (m, 4H), 0.50–0.45 (m, 4H); ^{13}C NMR (151 MHz, $CDCl_3$): δ 153.75, 151.81, 143.26, 139.12, 135.68, 130.85, 130.65, 129.78, 129.40, 128.81, 126.11, 122.77 122.42, 121.78, 121.41, 120.14, 110.12, 55.44, 44.57, 40.01, 29.56, 26.92, 23.29. HRMS (ESI) (*m/z*): $[M+H]^+$ calcd. for $C_{49}H_{45}Br_2N_4$: 847.20054, found: 847.20065.

1,1'-((2,7-dibromo-9*H*-fluorene-9,9-diyl)bis(octane-8,1-diyl))bis(2-phenyl-1*H*-benzo[*d*]imidazole (SI-2)

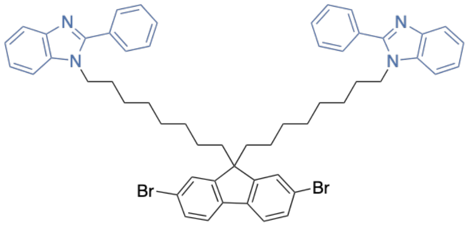

Compound **SI-2** was prepared following General Procedure A, using 2,7-dibromo-9,9-bis(8-bromooctyl)-9*H*-fluorene[2] (721 mg, 1.02 mmol), 2-phenylbenzimidazole (435 mg, 2.24 mmol), and NaH (97.9 mg, 4.08 mmol). Purification was accomplished by flash chromatography using a hexanes:ethyl acetate gradient, affording 711 mg (0.762 mmol, 75%) of a white solid. Data are as follows: ^{1}H NMR (600 MHz, $CDCl_3$): δ 7.83–7.80 (m, 2H), 7.68–7.66 (m, 4H), 7.49–7.47 (m, 8H), 7.44–7.41 (m, 4H), 7.39–7.36 (m, 2H), 7.31–7.28 (m, 4H), 4.16 (t, J = 7.7 Hz, 4H), 1.88–1.85 (m, 4H), 1.75–1.70 (m, 4H), 1.14–1.09 (m, 4H), 1.05–1.00 (m, 4H), 0.99–0.93 (m, 8H), 0.57–

0.48 (m, 4H); ^{13}C NMR (151 MHz, $CDCl_3$): δ 153.85, 152.52, 143.34, 139.23, 135.75, 130.94, 130.39, 129.77, 129.47, 128.79, 126.25, 122.74, 122.41, 121.64, 121.34, 120.15, 110.21, 55.77, 44.77, 40.21, 29.76, 29.73, 28.97, 28.89, 26.64, 23.61. HRMS (ESI) (*m/z*): $[M+H]^+$ calcd. for $C_{55}H_{57}Br_2N_4$: 931.29444, found: 931.29472.

1,1'-((2,7-dibromo-9*H*-fluorene-9,9-diyl)bis(decane-10,1-diyl))bis(2-phenyl-1*H*-benzo[*d*]imidazole (SI-3)

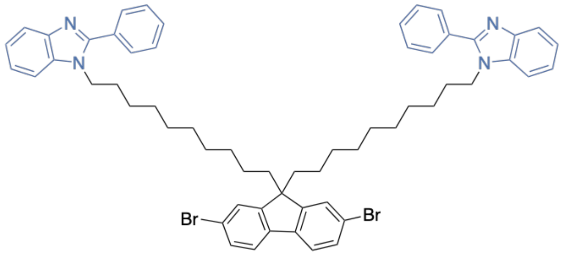

Compound **SI-3** was prepared following General Procedure A, using **SI-0** (778 mg, 1.02 mmol), 2-phenylbenzimidazole (435 mg, 2.24 mmol), and NaH (97.9 mg, 4.08 mmol). Purification was accomplished by flash chromatography using a hexanes:ethyl acetate gradient, affording 656 mg (0.663 mmol, 65%) of a white solid. Data are as follows: ^{1}H NMR (600 MHz, $CDCl_3$): δ 7.89–7.80 (m, 2H), 7.73–7.66 (m, 4H), 7.51–7.44 (m, 10H), 7.44–7.40 (br, 2H), 7.40–7.36 (m, 2H), 7.31–7.27 (m, 4H), 4.17 (t, J = 7.6 Hz, 4H), 1.94–1.88 (m, 4H), 1.79–1.71 (m, 4H), 1.22–1.10 (br, 8H), 1.09–0.98 (br, 16H), 0.63–0.53 (m, 4H); ^{13}C NMR (151 MHz, $CDCl_3$): δ 153.66, 152.46, 143.17, 139.04, 135.60, 130.74, 130.17, 129.60, 129.28, 128.63, 126.11, 122.59, 122.24, 121.47, 121.17, 119.94, 110.10, 55.65, 44.61, 40.07, 29.69, 29.62, 29.20, 29.16, 28.99, 28.82, 26.53, 23.54. HRMS (ESI) (*m/z*): $[M+H]^+$ calcd. for $C_{59}H_{65}Br_2N_4$: 987.35705, found: 987.35783.

5. Synthesis of functionalized conjugated polymers.

5.1 General Procedure B: Synthesis of Functionalized Polymers.

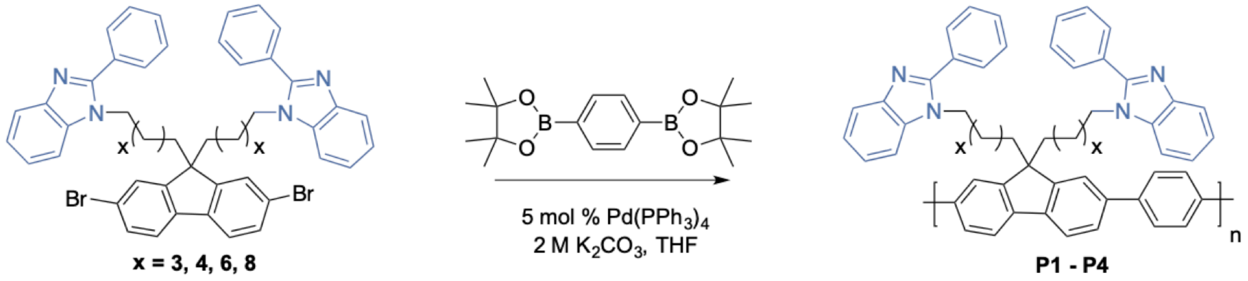

A microwave tube was loaded with bifunctional aromatic bromide (**SI-1 - SI-3**) (0.100 mmol, 1.00 equiv.), 1,4-bis(4,4,5,5-tetramethyl-1,3,2-dioxaborolan-2-yl)benzene (0.105 mmol, 1.05 equiv.), 2 M K_2CO_3 (1.0 mL, 20 equiv.) in water, and THF (1.0 mL). The mixture was sparged with nitrogen, and 0.70 mL of a $Pd(PPh_3)_4$/THF stock solution (5 mol %) was added via syringe. The mixture was stirred vigorously and heated conventionally at 70 °C for 15 h. After this time, the reaction was allowed to cool leaving a solid gelled material. The mixture was precipitated into methanol and collected via filtration. The residual solid was loaded into an extraction thimble and washed with methanol (8 h), acetone (4 h), and hexanes (4 h). The polymer was dried in vacuo.

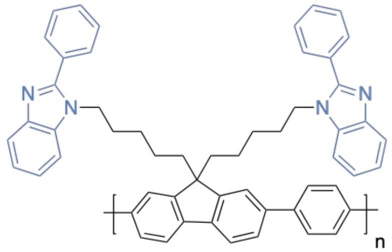

P1: Compound **P1** was prepared following General Procedure B, using **SI-1** (84.9 mg, 0.100 mmol) affording 32.4 mg (42%) of a light brown solid. Data are as follows: ^{1}H NMR (600 MHz, $CDCl_3$): δ 7.87–7.77 (br, 4H), 7.75–7.66 (br, 5H), 7.63–7.59 (br, 4H), 7.57–7.50 (br, 2H), 7.49–7.31 (br, 6H), 7.28–7.20 (m, 7H), 4.10–4.00 (br, 4H), 2.25–1.79 (br, 4H), 1.67–1.57 (br, 4H), 1.16–1.00 (br, 4H), 0.85–0.57 (br, 4H). GPC (160 °C, 1,2,4-trichlorobenzene) M_n = 8.5 kg mol^{-1}, $Đ$ = 3.7.

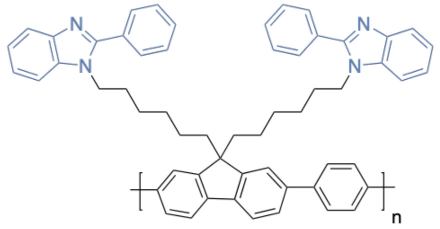

P2: Compound **P2** was prepared following previously reported procedures,[3] using 1,1'-((2,7-dibromo-9*H*-fluorene-9,9-diyl)bis(hexane-6,1-diyl))bis(2-phenyl-1*H*-benzo[*d*]imidazole) (87.7 mg, 0.100 mmol) affording 48.5 mg (61%) of a light brown

solid. NMR was consistent with what was previously reported. GPC (160 °C, 1,2,4-trichlorobenzene) M_n = 10.6 kg mol^{-1}, $Đ$ = 2.6.

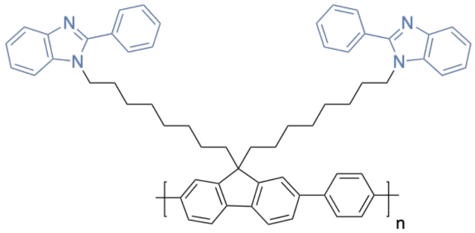

P3: Compound **P3** was prepared following General Procedure B, using **SI-2** (93.2 mg, 0.100 mmol) affording 41.1 mg (48%) of a light brown solid. Data are as follows: ^{1}H NMR (600 MHz, $CDCl_3$): δ 7.86–7.73 (br, 7H), 7.72–7.57 (br, 8H), 7.50–7.40 (br, 6H), 7.36–7.31 (br, 2H), 7.29–7.20 (br, 5H), 4.19–4.07 (m, 4H), 2.45–1.93 (br, 4H), 1.77–1.65 (m, 4H), 1.16–0.97 (br, 16H), 0.89–0.60 (br, 4H). GPC (160 °C, 1,2,4-trichlorobenzene) M_n = 7.2 kg mol^{-1}, $Đ$ = 2.4.

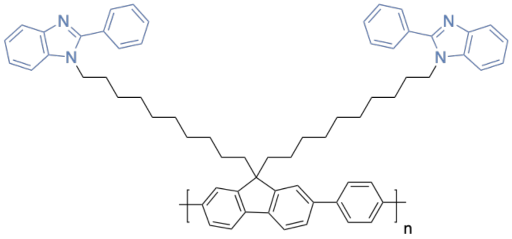

P4: Compound **P4** was prepared following General Procedure B, using **SI-3** (98.9 mg, 0.100 mmol) affording 38.2 mg (42%) of a grey solid. Data are as follows: ^{1}H NMR (600 MHz, $CDCl_3$): δ 7.89–7.73 (br, 7H), 7.73–7.58 (br, 8H), 7.48–7.41 (br, 6H), 7.37–7.31 (br, 2H), 7.30–7.25 (br, 5H), 4.22–4.08 (m, 4H), 2.42–1.88 (br, 4H), 1.76–1.69 (m, 4H), 1.16–0.97 (br, 24H), 0.86–0.63 (br, 4H). GPC (160 °C, 1,2,4-trichlorobenzene) M_n = 19.9 kg mol^{-1}, $Đ$ = 2.6.

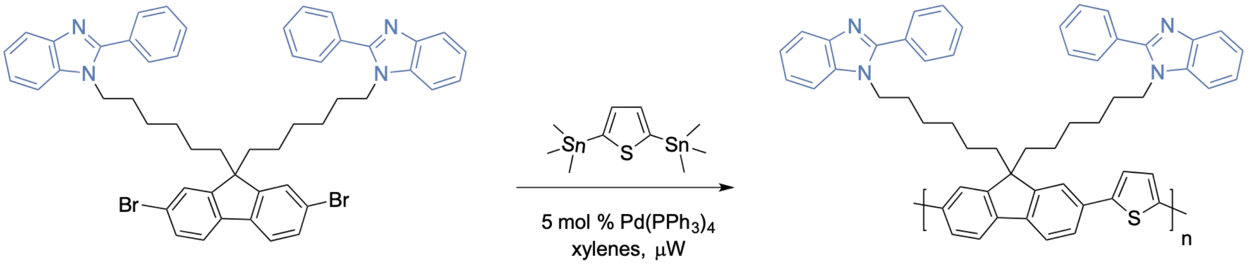

P5: A microwave tube was loaded with 1,1’-((2,7-dibromo-9*H*-fluorene-9,9-diyl)bis(hexane-6,1-diyl))bis(2-phenyl-1*H*-benzo[*d*]imidazole)[3] (78.9 mg, 0.0900 mmol) and 2,5-bis(trimethylstannyl)thiophene (38.7 mg, 0.0945 mmol). The tube was brought inside a glove box, and 619 μL of a $Pd(PPh_3)_4$/xylenes stock solution (5 mol %) was added. The tube was sealed and subjected to 140 °C for 10 min in a microwave reactor with stirring. After this time, the reaction was allowed to cool leaving a

solid gelled material. The mixture was precipitated into methanol and collected via filtration. The residual solid was loaded into an extraction thimble and washed with methanol (8 h), acetone (4 h), and hexanes (4 h). The polymer was dried in vacuo affording 62.3 mg (86%) of a brown solid. Data are as follows: ^{1}H NMR (600 MHz, $CDCl_3$): δ 7.92–7.50 (br, 12H), 7.47–7.30 (br, 8H), 7.26–7.11 (br, 6H), 4.24–3.85 (br, 4H), 2.14–1.78 (br, 4H), 1.67–1.54 (br, 4H), 1.19–0.87 (br, 8H), 0.77–0.54 (br, 4H). GPC (160 °C, 1,2,4-trichlorobenzene) M_n = 9.9 kg mol^{-1}, $Đ$ = 3.0.

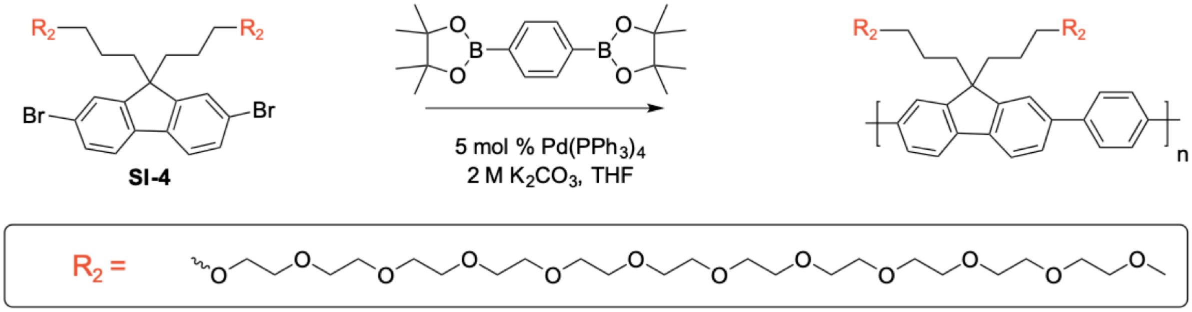

P6: Compound **P6** was prepared following previously reported procedures,[4] **SI-4** (62.7 mg, 0.0490 mmol) affording 52.7 mg (79%) of a light green solid. NMR was consistent with what was previously reported. GPC (160 °C, 1,2,4-trichlorobenzene) M_n = 11.5 kg mol^{-1}, $Đ$ = 2.3.

6. NMR Spectra

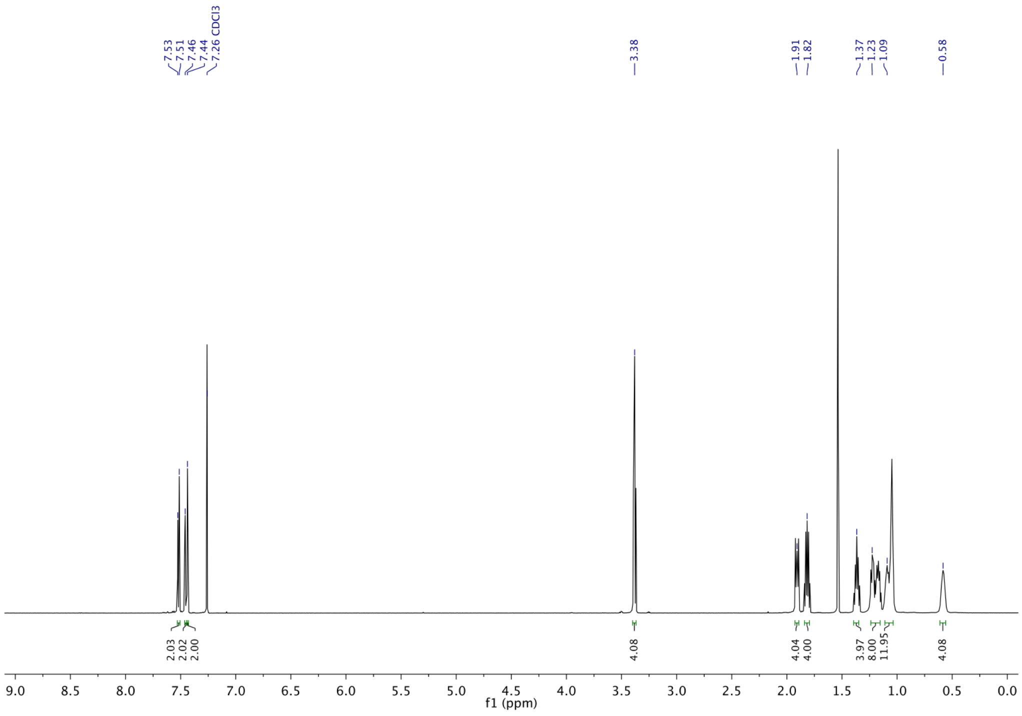

Fig. S46 ^{1}H NMR (600 MHz, $CDCl_3$) of 2,7-dibromo-9,9-bis(10-bromodecyl)-9*H*-fluorene (**SI-0**).

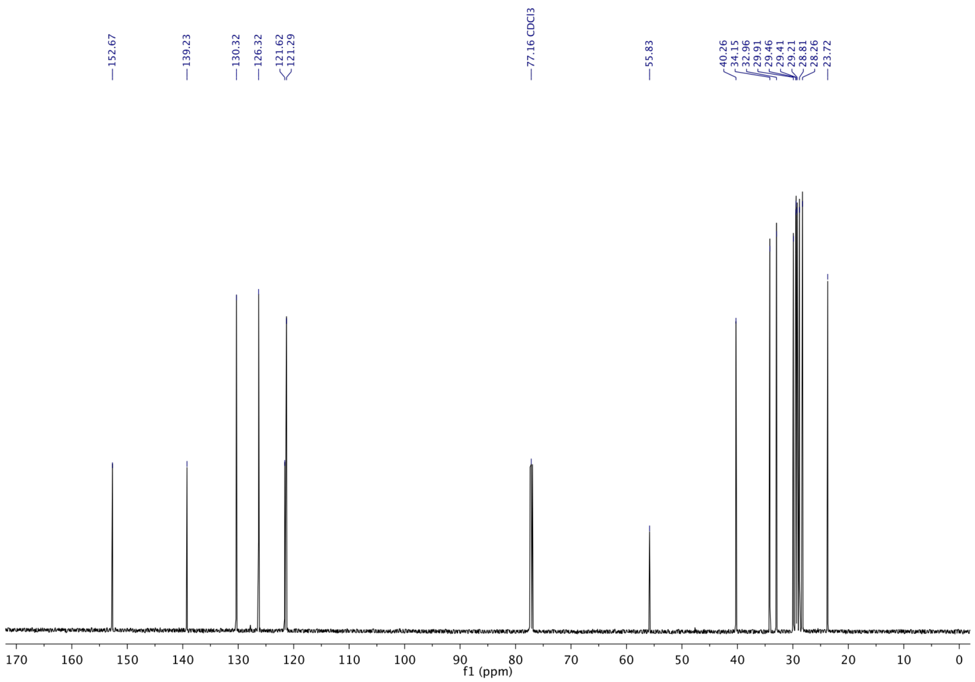

Fig. S47 ^{13}C NMR (151 MHz, $CDCl_3$) of 2,7-dibromo-9,9-bis(10-bromodecyl)-9*H*-fluorene (**SI-0**).

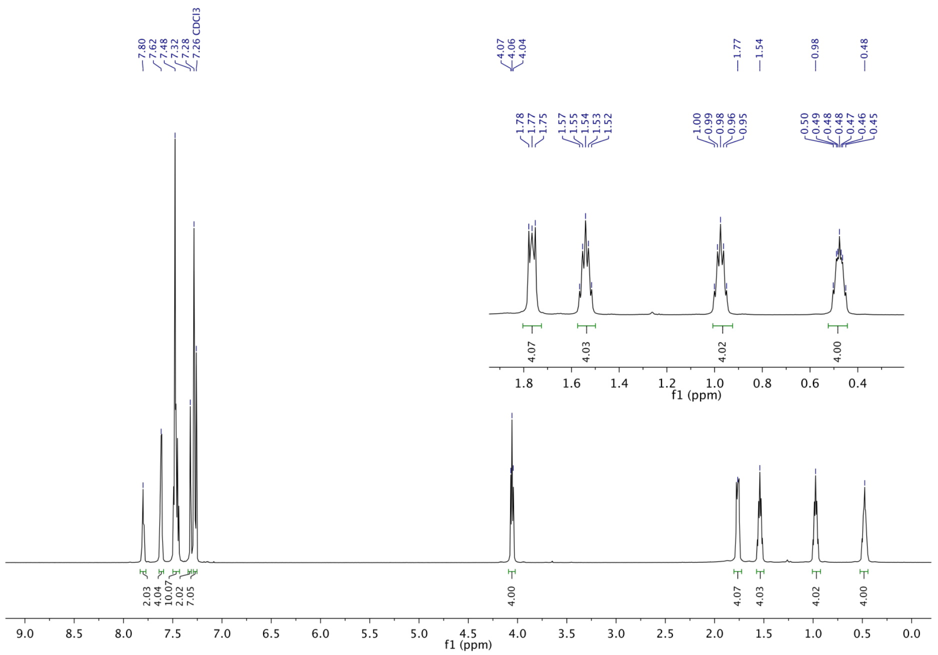

Fig. S48 ^{1}H NMR (600 MHz, $CDCl_3$) of 1,1'-((2,7-dibromo-9*H*-fluorene-9,9-diyl)bis(pentane-5,1-diyl))bis(2-phenyl-1*H*-benzo[*d*]imidazole) (**SI-1**).

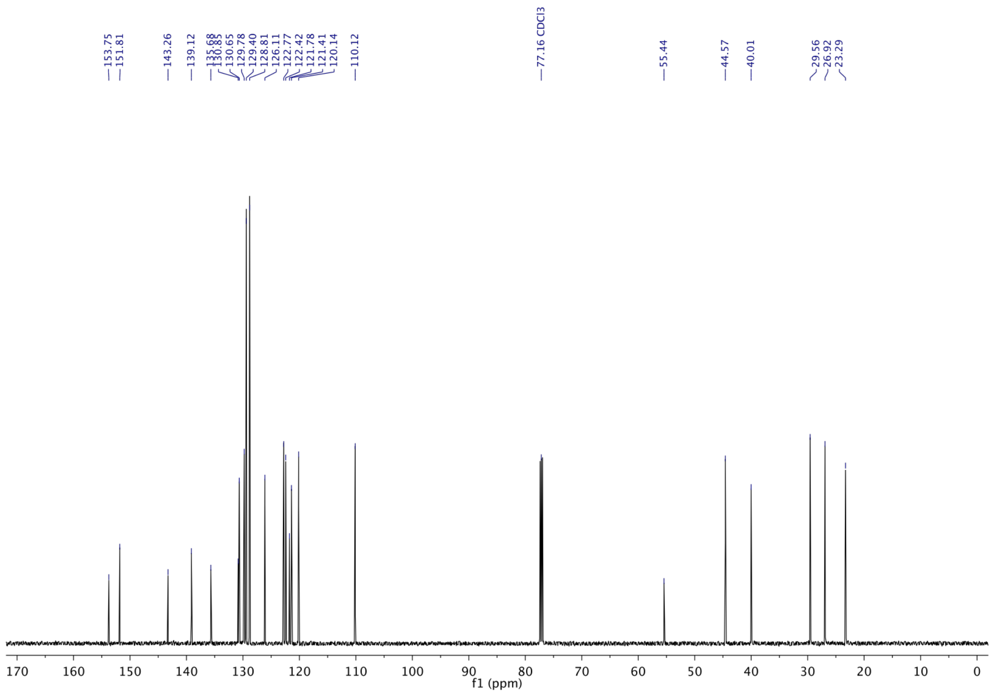

Fig. S49 ^{13}C NMR (151 MHz, $CDCl_3$) of 1,1'-((2,7-dibromo-9*H*-fluorene-9,9-diyl)bis(pentane-5,1-diyl))bis(2-phenyl-1*H*-benzo[*d*]imidazole) (**SI-1**).

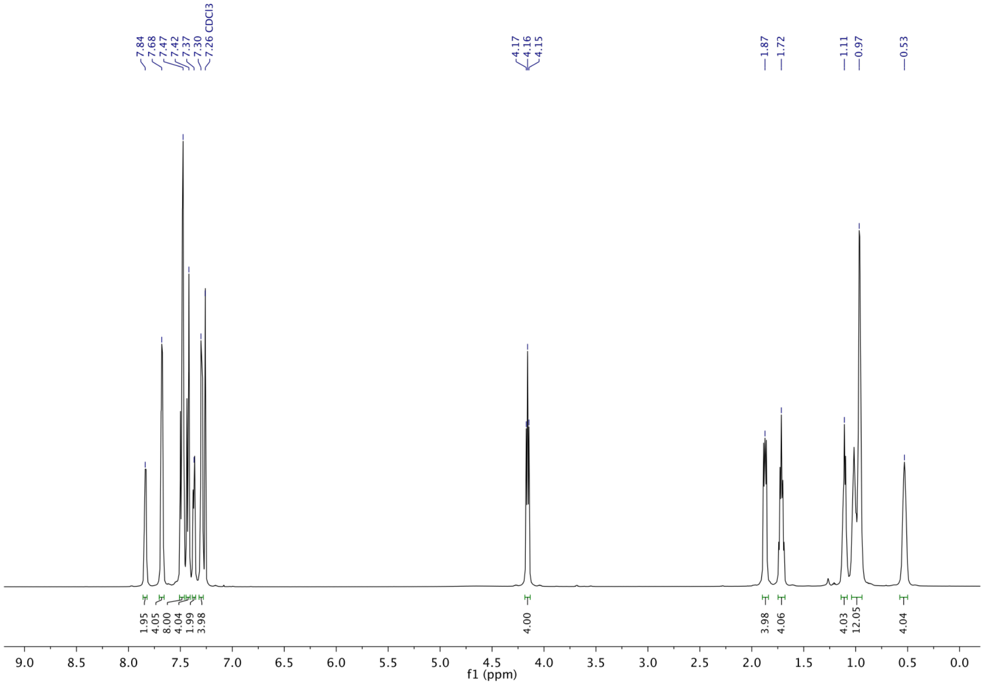

Fig. S50 ^{1}H NMR (600 MHz, $CDCl_3$) of 1,1'-((2,7-dibromo-9*H*-fluorene-9,9-diyl)bis(octane-8,1-diyl))bis(2-phenyl-1*H*-benzo[*d*]imidazole (**SI-2**).

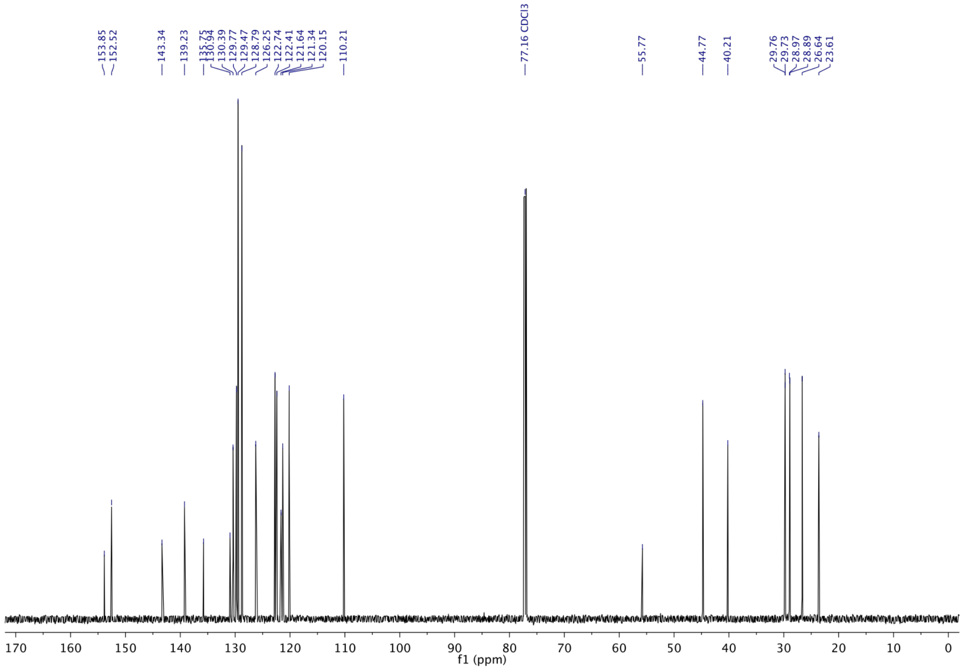

Fig. S51 ^{13}C NMR (151 MHz, $CDCl_3$) of 1,1'-((2,7-dibromo-9*H*-fluorene-9,9-diyl)bis(octane-8,1-diyl))bis(2-phenyl-1*H*-benzo[*d*]imidazole (**SI-2**).

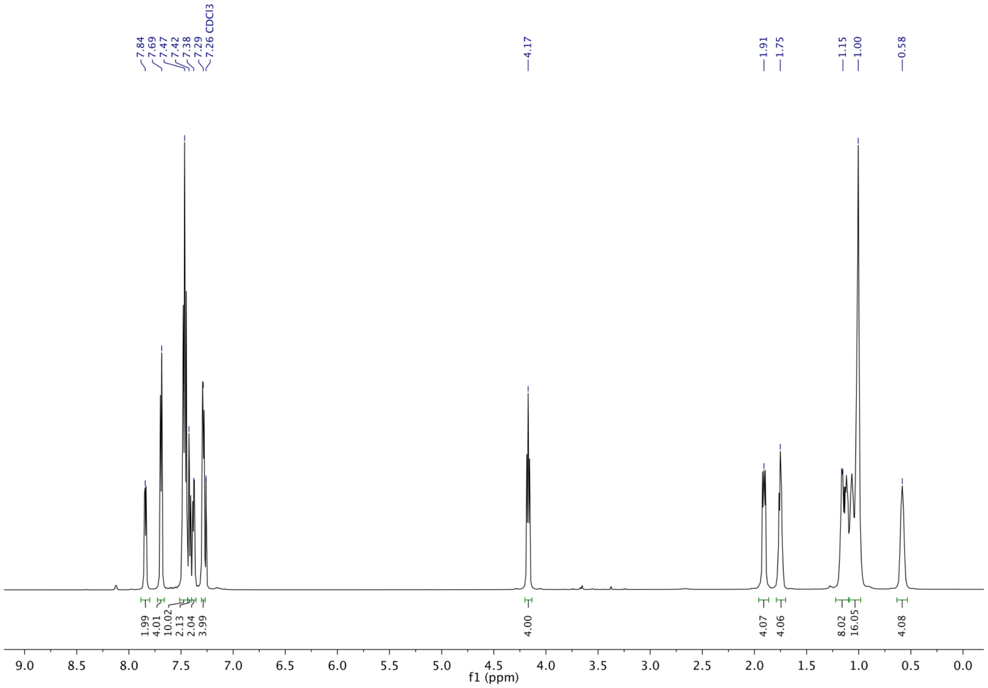

Fig. S52 ^{1}H NMR (600 MHz, $CDCl_3$) of 1,1'-((2,7-dibromo-9*H*-fluorene-9,9-diyl)bis(decane-10,1-diyl))bis(2-phenyl-1*H*-benzo[*d*]imidazole (**SI-3**).

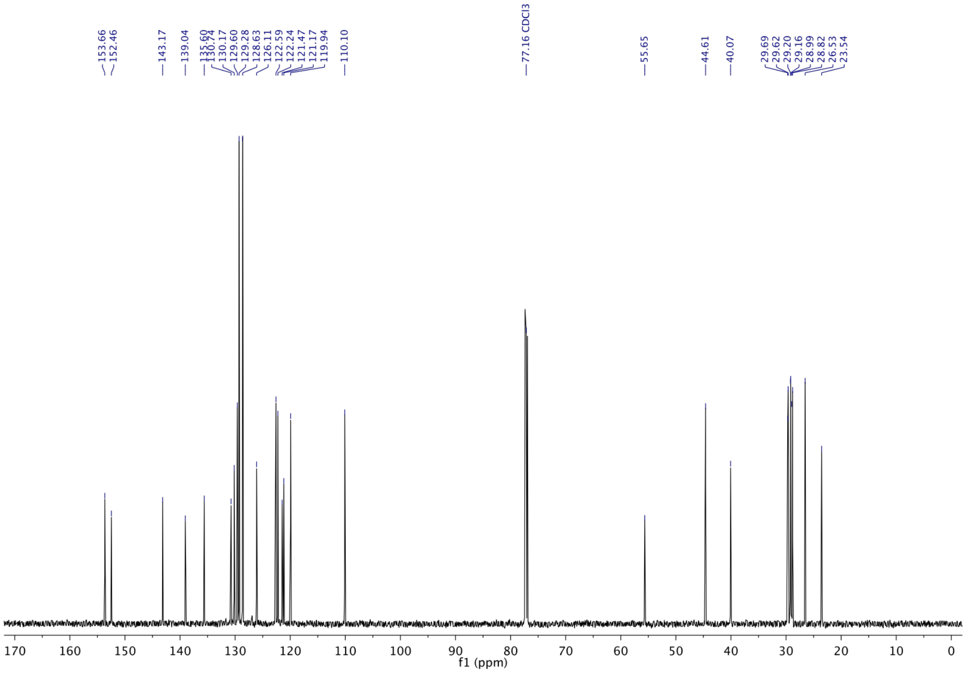

Fig. S53 ^{13}C NMR (151 MHz, $CDCl_3$) of 1,1'-((2,7-dibromo-9*H*-fluorene-9,9-diyl)bis(decane-10,1-diyl))bis(2-phenyl-1*H*-benzo[*d*]imidazole (**SI-3**).

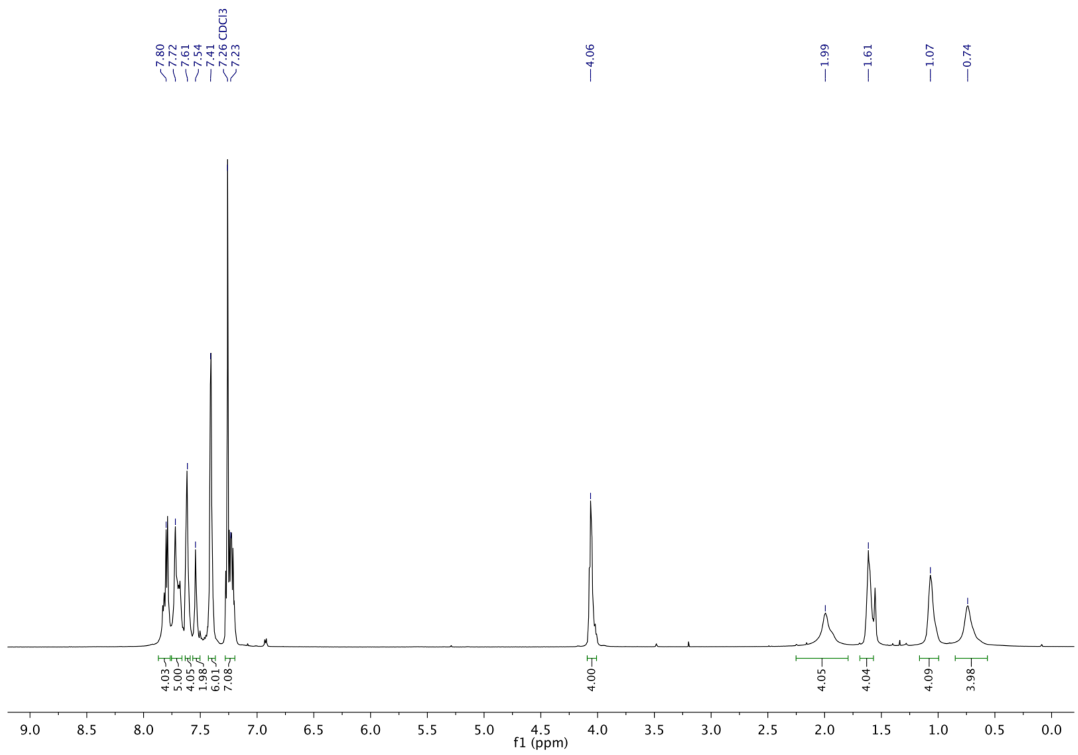

Fig. S54 ^{1}H NMR (600 MHz, $CDCl_3$) of **P1.**

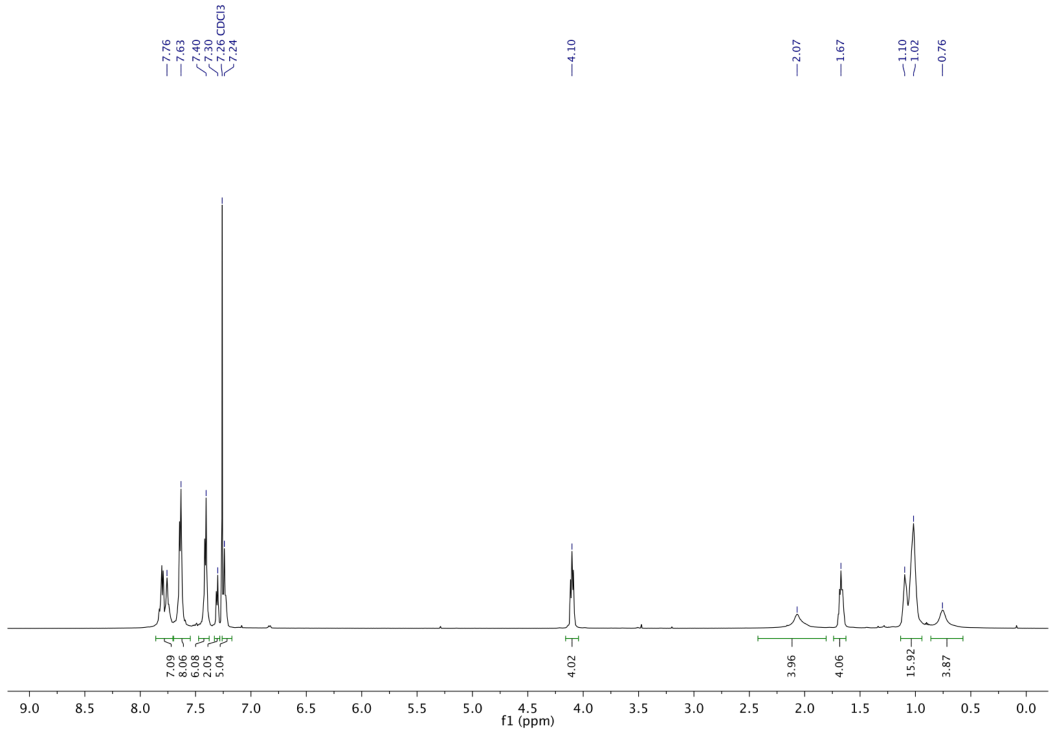

Fig. S55 ^{1}H NMR (600 MHz, $CDCl_3$) of **P3.**

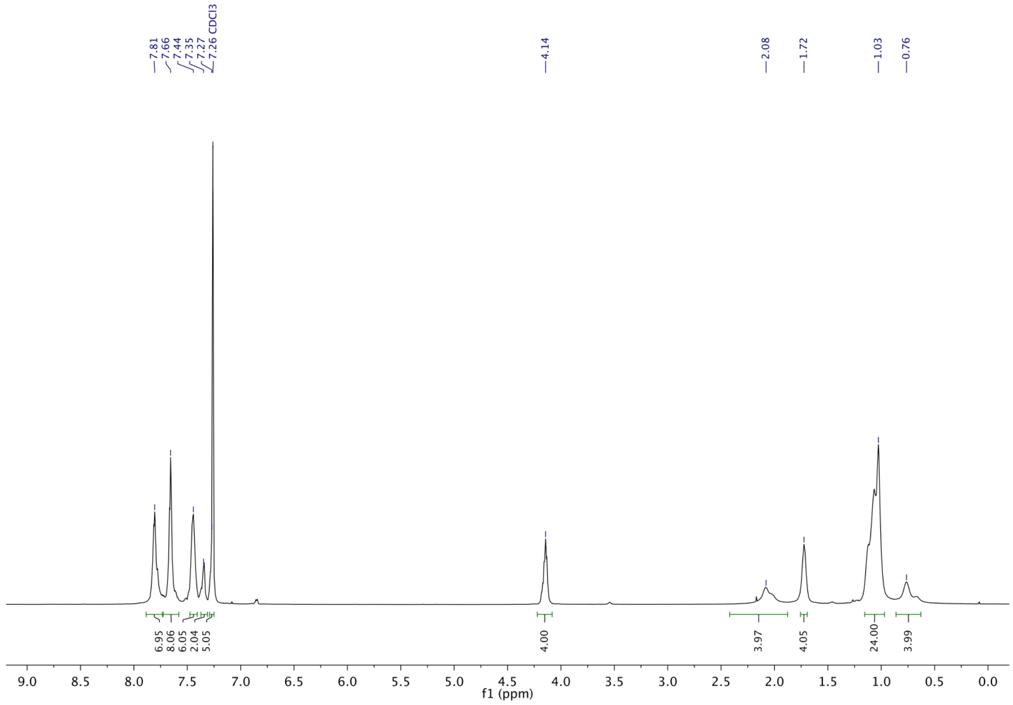

Fig. S56 ^{1}H NMR (600 MHz, $CDCl_3$) of **P4.**

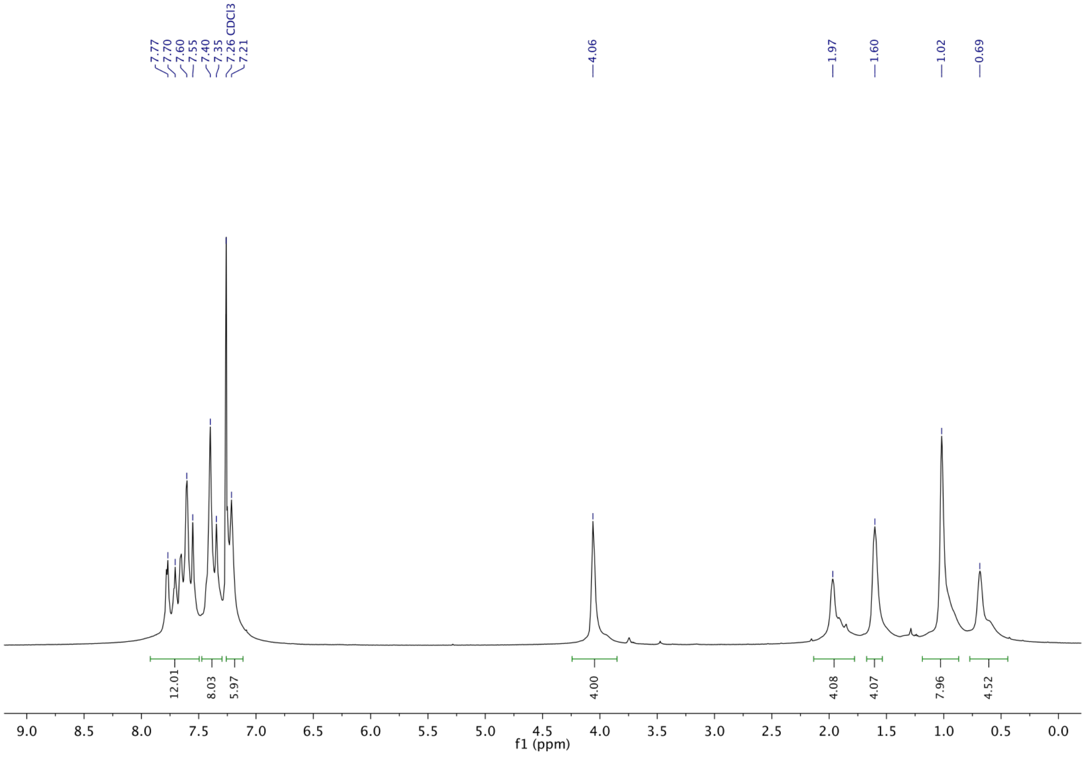

Fig. S57 ^{1}H NMR (600 MHz, $CDCl_3$) of **P5.**

7. References.

1. C. -S. Wu, C. -Y. Chou, Y. Chen, *J. Mater. Chem. C*, 2014, **2**, 6665.
2. K. Kim, M. Suh, J. Choi, D. Lee, Y. Kim, S. H. Cheong, D. Kim, D. Y. Jeon, *Adv. Funct. Mater.*, 2015, **25**, 7450.
3. G. Saikia, P. K. Iyer, *Macromolecules*, 2011, **44***,* 3753.
4. B. S. Gaylord, J. M. Wheeler, G. P. Bartholomew, Y. Liang, J. W. Hong, W. H. Huisman, F. P. Uckert, L. T. Tran, A. C. V. Palmer, T. Nguyen, R. Baldocchi, US 2011/0257374 A1, 2011/01/19, 2011**.**
